# Dynamic associations between glucose and ecological momentary cognition in Type 1 Diabetes

**DOI:** 10.1038/s41746-024-01036-5

**Published:** 2024-03-18

**Authors:** Z. W. Hawks, E. D. Beck, L. Jung, L. M. Fonseca, M. J. Sliwinski, R. S. Weinstock, E. Grinspoon, I. Xu, R. W. Strong, S. Singh, H. P. A. Van Dongen, M. R. Frumkin, J. Bulger, M. J. Cleveland, K. Janess, Y. C. Kudva, R. Pratley, M. R. Rickels, S. R. Rizvi, N. S. Chaytor, L. T. Germine

**Affiliations:** 1 Institute for Technology in Psychiatry, McLean Hospital, Belmont, MA USA; 2 Department of Psychiatry, Harvard Medical School, Boston, MA USA; 3 Department of Psychology, University of California Davis, Davis, CA USA; 4 Elson S. Floyd College of Medicine, Washington State University, Spokane, WA USA; 5 Programa Terceira Idade (PROTER, Old Age Research Group), Department and Institute of Psychiatry, University of São Paulo School of Medicine, São Paulo, Brazil; 6 Department of Human Development and Family Studies, Center for Healthy Aging, Pennsylvania State University, State College, PA USA; 7 SUNY Upstate Medical University, Syracuse, NY USA; 8 Department of Psychology, University of Notre Dame, Notre Dame, IN USA; 9 The Many Brains Project, Belmont, MA USA; 10 Sleep and Performance Research Center & Department of Translational Medicine and Physiology, Washington State University, Spokane, WA USA; 11 Department of Psychological & Brain Sciences, Washington University in St. Louis, St. Louis, MO USA; 12 Department of Psychiatry, Massachusetts General Hospital, Boston, MA USA; 13 Department of Human Development, Washington State University, Pullman, WA USA; 14 Jaeb Center for Health Research, Tampa, FL USA; 15 Division of Endocrinology, Diabetes and Nutrition, Mayo Clinic, Rochester, MN USA; 16 AdventHealth Translational Research Institute, Orlando, FL USA; 17 University of Pennsylvania Perelman School of Medicine, Philadelphia, PA USA

**Keywords:** Type 1 diabetes, Human behaviour

## Abstract

Type 1 diabetes (T1D) is a chronic condition characterized by glucose fluctuations. Laboratory studies suggest that cognition is reduced when glucose is very low (hypoglycemia) and very high (hyperglycemia). Until recently, technological limitations prevented researchers from understanding how naturally-occurring glucose fluctuations impact cognitive fluctuations. This study leveraged advances in continuous glucose monitoring (CGM) and cognitive ecological momentary assessment (EMA) to characterize dynamic, within-person associations between glucose and cognition in naturalistic environments. Using CGM and EMA, we obtained intensive longitudinal measurements of glucose and cognition (processing speed, sustained attention) in 200 adults with T1D. First, we used hierarchical Bayesian modeling to estimate dynamic, within-person associations between glucose and cognition. Consistent with laboratory studies, we hypothesized that cognitive performance would be reduced at low and high glucose, reflecting cognitive vulnerability to glucose fluctuations. Second, we used data-driven lasso regression to identify clinical characteristics that predicted individual differences in cognitive vulnerability to glucose fluctuations. Large glucose fluctuations were associated with slower and less accurate processing speed, although slight glucose elevations (relative to person-level means) were associated with faster processing speed. Glucose fluctuations were not related to sustained attention. Seven clinical characteristics predicted individual differences in cognitive vulnerability to glucose fluctuations: age, time in hypoglycemia, lifetime severe hypoglycemic events, microvascular complications, glucose variability, fatigue, and neck circumference. Results establish the impact of glucose on processing speed in naturalistic environments, suggest that minimizing glucose fluctuations is important for optimizing processing speed, and identify several clinical characteristics that may exacerbate cognitive vulnerability to glucose fluctuations.

## Introduction

Type 1 diabetes (T1D) is a chronic condition characterized by elevated glucose and increased glucose variability^1,2^. Among individuals with T1D, elevated glucose and increased glucose variability are related to adverse health outcomes, including mild neurocognitive disorder, dementia, and microvascular complications^3–7^. Laboratory studies indicate that very low (hypo) and, to a lesser extent, very high (hyper) glycemia both impair cognitive performance in those with T1D^8–15^. It remains unclear how continuous variation in glucose impacts cognitive fluctuations in naturalistic environments, with implications for everyday safety (e.g., driving, rapid decision-making)^16^. The magnitude of naturalistic cognitive fluctuations also shows promise to stratify individuals based on clinical risk^17^.

Until recently, technological limitations prevented researchers from understanding how moment-to-moment fluctuations in glucose impact moment-to-moment fluctuations in cognition in naturalistic environments. New advances in cognitive ecological momentary assessment (EMA) and continuous glucose monitoring (CGM) enable high-frequency, high-quality data collection within individuals over time. In cognitive EMA, participants complete ultra-brief cognitive tasks several times each day using smartphone devices. Cognitive EMA tasks are validated for repeated, remote, digital administration, and they reliably capture between- and within-person cognitive variation^18–20^. Just as EMA supports reliable and valid cognitive assessment, CGM devices sample glucose frequently (e.g., every five minutes), generating intensive longitudinal time series data of sufficient quality to enable medical decision-making and automated insulin delivery^21,22^. Together, EMA and CGM timeseries data provide new opportunities to improve understanding of within-person, naturalistic associations between glucose and cognition in T1D. In one of the few studies to date to combine EMA and CGM, overnight glucose variability and hypoglycemia exposure predicted next-day fluctuations in sustained attention^23^. To our knowledge, moment-to-moment dynamic associations between glucose and cognition have not been examined. Addressing this gap is critical to clarify when and for whom glucose fluctuations predict cognitive impairment, informing the development of empirically supported, person- and context-specific recommendations for diabetes self-management that maximize glycemic control and cognitive performance.

**Fig. 1:**
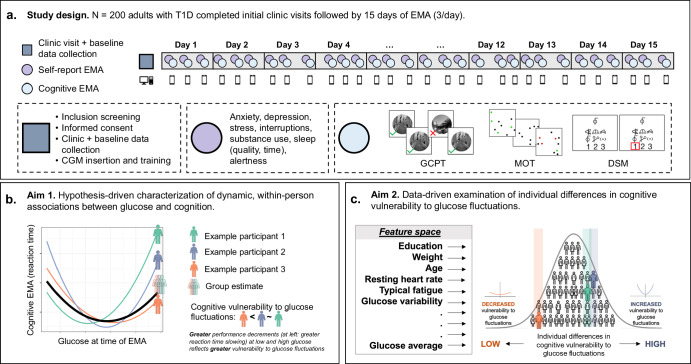
Schematic of study design, aims, and methods. Results are summarized in Figs 3–5. **a** Adults with type 1 diabetes (T1D) completed initial clinic visits and baseline cognitive data collection followed by 15 days of ecological momentary assessment (EMA). **b** Analyses characterized dynamic, within-person associations between glucose and cognition for the full sample (group estimate; thick black line) and each participant (individual estimates; example participants 1–3 in green, purple, and orange). U-shaped curves for speeded outcomes (depicted below) indicate slower reaction time at low and high glucose, whereas inverted curves for accuracy outcomes indicate reduced accuracy at low and high glucose. Steeper curves (e.g., example participants 1-2, shaded green and purple) indicate greater cognitive vulnerability to glucose fluctuations, whereas shallower curves (e.g., example participant 3, shaded orange) indicate reduced cognitive vulnerability to glucose fluctuations. **c** Data-driven analyses identified strong person-level predictors of individual differences in cognitive vulnerability to glucose fluctuations. Strong predictors were selected from a feature space that included 58 clinical, physiological, and demographic variables. *Gradual onset continuous performance test [GCPT], multiple object tracking [MOT], digit-symbol matching [DSM]*.

**Fig. 2:**
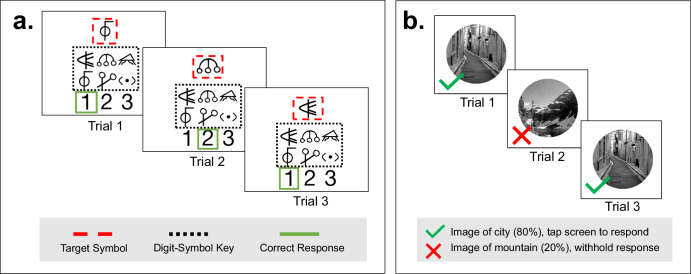
Example trials for Digit-Symbol Matching (DSM) and Gradual Onset Continuous Performance Test (GCPT). **a** DSM: participants were presented with a target symbol and a digit-symbol pairing key. They used their touchscreen to press the digit that was paired with the target symbol in the key. There was no response deadline, and each EMA session lasted 30 seconds. **b** GCPT: participants viewed a circular, grayscale image of a city or mountain. They were instructed to press their touchscreen device when the image depicted a city and withhold a response when the image depicted a mountain. Each EMA session lasted 60 seconds and consisted of 75 trials. *Legend items were not visible during administration*.

To these ends, the present study characterized dynamic, within-person associations between glucose and cognition (Fig. 1). Using CGM and EMA, we obtained intensive longitudinal glucose and cognitive timeseries in 200 adults with T1D (Table 1). Cognitive tasks measured processing speed (digital symbol matching [DSM]) and sustained attention (gradual onset continuous performance test [GCPT]) (Fig. 2). In Aim 1, we evaluated within-person associations between glucose and cognition. We hypothesized that cognitive performance would be reduced at low and high glucose, reflecting cognitive vulnerability to glucose fluctuations (hypothesis 1 [H1]). We also expected to observe individual differences in cognitive vulnerability to glucose fluctuations (hypothesis 2 [H2]). In Aim 2, we identified clinical characteristics that predicted individual differences in cognitive vulnerability to glucose fluctuations. Successful execution of study aims promises to establish the short-term impact of glucose fluctuations on cognition in naturalistic environments, inform person- and context-specific recommendations for diabetes self-management, and identify a limited number of large-effect clinical characteristics that exacerbate cognitive vulnerability to glucose fluctuations.

**Table 1:** Participant characteristics

Variable		Statistic
Categorical	Level	% (*N*)
Education	High school	6 (12)
	Technical school	17 (34)
	College degree	55 (110)
	Master’s degree	15.5 (31)
	Graduate degree (e.g., PhD, JD, MD)	5 (10)
	Not sure or not reported	1.5 (3)
Ethnicity	Hispanic or Latinx	6.5 (13)
	Not Hispanic or Latinx	92 (184)
	Not sure or not reported	1.5 (3)
Gender	Female	53.5 (107)
	Male	44.5 (89)
	Nonbinary	0.5 (1)
	Not sure or not reported	1.5 (3)
Race	Asian	1 (2)
	Black or African American	5.5 (11)
	Hawaiian or Pacific Islander	0.5 (1)
	White	86 (172)
	Multiracial	2 (4)
	Not sure or not reported	5 (10)
gluQuartile (mg/dL)	1st: 138.02	25 (50)
	2nd: 162.5	25 (50)
	3rd: 186.54	25 (50)
	4th: 242.28	25 (50)
SevereHypoEvents	0	41 (82)
	1	17 (34)
	2	10 (20)
	3	7.5 (15)
	4	3.5 (7)
	5–10	11 (22)
	10+	10 (20)
**Continuous**		**M (SD, Range)**
Age (years)	–	45.7 (15.6, 18–84)
HbA1c (mmol/mol)	–	7.5 (1.3, 5.1–12.3)
gluMean (mg/dL)	–	182.3 (44.3, 115–380)
gluSD (mg/dL)	–	65.6 (18.8, 24–117)
gluCV (%)	–	36.1 (7.3, 11–68)
gluInRange70_180 (%)	–	54.6 (20, 0–97)
gluBelow70 (%)	–	2.9 (3.6, 0–26.5)
gluBelow54 (%)	–	0.9 (1.8, 0–16.9)
gluAbove180 (%)	–	42.5 (20.8, 2–100)
gluAbove250 (%)	–	18.4 (18.5, 0–97)

% (*N*) percent (count), *M (SD*) mean (standard deviation), *gluQuartile* mean glucose within each quartile, *SevereHypoEvents* number of lifetime severe hypoglycemic events, *HbA1c* hemoglobin A1c test, gluMean, gluSD, *gluCV* participant-level glucose means, standard deviations, and coefficients of variation, respectively, during the study period; gluInRange70_180 = % time glucose was in range (70–180 mg/dL); gluBelow70, gluBelow54 = % time glucose was below 70 mg/dL and 54 mg/dL, respectively; gluAbove180, gluAbove250 = % time glucose was above 180 mg/dL and 250 mg/dL, respectively.

## Results

### Demographic characteristics and descriptive statistics

Table 1 reports participant characteristics, and Table 2 provides descriptive statistics and multilevel reliability estimates for cognitive EMA variables. After data cleaning, the analysis sample (*n* = 190) did not differ from the full sample (N = 200) with respect to any of the following: age, gender, race, ethnicity, educational attainment, hemoglobin A1c, CGM summary statistics (glucose mean, standard deviation, coefficient of variation), CGM percent time in range (70–180 mg/dL, below 70 mg/dL, below 54 mg/dL, above 180 mg/dL, above 250 mg/dL), or number of lifetime severe hypoglycemic events (ps > 0.05).

**Table 2:** Cognitive descriptive statistics and multilevel reliability estimates

Outcome	Mean (SD)	Range	WPR	BPR	N (T)
DSM RT	952 (218)	612–2092	0.57	0.99	190 (38.2)
DSM num correct	43 (7.6)	20.1–58.1	0.58	0.99	190 (38.2)
GCPT RT	822 (87)	539–1014	0.76	0.99	190 (38.1)
GCPT d-prime	2.47 (0.65)	0.51–3.68	0.55	0.98	190 (38.1)

Between-person reliability (BPR) and within-person reliability (WPR) estimates were consistent with prior research^62,93,94^. *SD* standard deviation, *WPR* within-person reliability, *BPR* between person reliability, *N* number of participants, *T* number of EMAs completed, *MOT* multiple object tracking, *DSM* digit symbol, *GCPT* gradual onset continuous performance test, *RT* reaction time, *num* number.

### Aim 1: Processing speed, but not sustained attention, was vulnerable to glucose fluctuations

We used hierarchical Bayesian modeling to estimate dynamic, within-person associations between glucose and cognition. To capture cognitive vulnerability to low and high glucose fluctuations, we modeled glucose using quadratic polynomials. Quadratic terms for glucose were examined for the full sample (group estimates; in Methods, γ_20_) as well as each participant (individual estimates; in Methods, *u*_2*j*_). To evaluate the impact of potential observation and/or sample selection biases, we ran models across three EMA completion cutoffs (≥50%, ≥66%, ≥80%). We focus interpretation on results that were significant across all completion cutoffs.

**Fig. 3:**
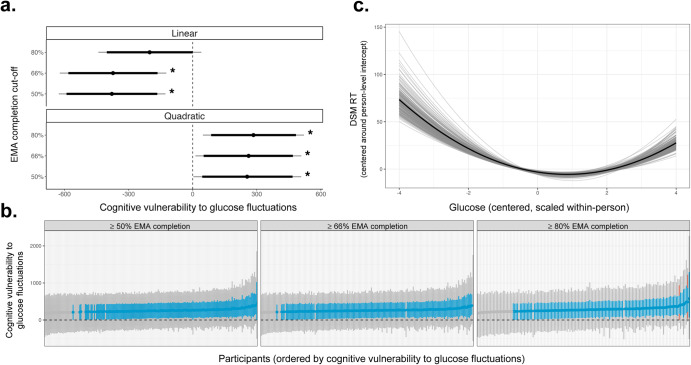
Group and individual estimates of cognitive vulnerability to glucose fluctuations. Counter-clockwise from top left: **a** Group estimates and credible intervals (CIs) for linear and quadratic terms relating glucose to DSM RT (x-axis), evaluated across EMA completion cut-offs (y-axis). 90% CIs are in black, and 95% CIs are in gray. Significant effects (marked by asterisks) were evaluated with respect to 95% CIs. Quadratic terms were significant across all EMA completion cut-offs, indicating cognitive vulnerability to glucose fluctuations. **b** Variation in individual estimates of cognitive vulnerability to glucose fluctuations for DSM RT. Cognitive vulnerability to glucose fluctuations (y-axis) is visualized for each participant (x-axis) across EMA completion cutoffs (panels). CIs illustrate different levels of uncertainty (95%, 90%, 66%) around individual estimates. Gray lines show 95% CIs, red lines show 90% CIs that do not overlap zero, and blue lines show 66% CIs that do not overlap zero. Most CIs are shaded blue but not red, suggesting moderate to high (66–90%) probability that a given individual exhibited cognitive vulnerability to glucose fluctuations. **c** Model-implied predictions relating glucose (x-axis) to DSM RT (y-axis) in ≥66% EMA completion data. Group predictions (based on **a**) are represented by the thick black line, and individual predictions (based on **b**) are represented by thin gray lines, where one gray line = one participant. DSM RT was slower at low and high glucose, reflecting cognitive vulnerability to glucose fluctuations. Variation in thin gray lines reflects individual differences in cognitive vulnerability to glucose fluctuations.

Consistent with H1, group estimates of cognitive vulnerability to glucose fluctuations were significant for DSM (Fig. 3a). Specifically, large glucose fluctuations were associated with slower and less accurate DSM performance. This pattern remained significant across all EMA completion cutoffs for DSM reaction time (RT). It was marginally significant using only the ≥66% EMA completion cutoff for DSM accuracy. Table 3 reports results using the ≥66% EMA completion cutoff, and supplementary materials (Supplementary Table 1, Supplementary Fig. 1) report results across all EMA completion cutoffs. Inconsistent with H1, group estimates of cognitive vulnerability to glucose fluctuations were not significant for GCPT. Results suggest that processing speed (DSM) may be more vulnerable to glucose fluctuations than sustained attention (GCPT).

**Table 3:** Estimates of within-person associations between glucose and cognition

Term	Estimate	SE	CIL	CIU
**DSM accuracy**				
(Intercept)	21.514	0.283	21.056	21.993
Glucose	9.786	2.585	5.521	14.049
Glucose^2^	−4.406	2.642	−8.701	−0.024
sd_(Intercept).participant	3.804	–	–	–
sd_Glucose.participant	3.862	–	–	–
sd_Glucose^2^.participant	3.695	–	–	–
cor_(Intercept).Glucose.particiant	0.297	–	–	–
cor_(Intercept).Glucose^2^.participant	0.119	–	–	–
cor_Glucose.Glucose^2^.participant	−0.058	–	–	–
sd_Observation.Residual	2.575	–	–	–
**DSM RT**				
(Intercept)	955.155	16.433	927.862	981.908
Glucose	−373.036	128.264	−582.097	−164.594
Glucose^2^	261.336	127.639	51.143	468.944
sd_(Intercept).participant	224.063	–	–	–
sd_Glucose.participant	312.381	–	–	–
sd_Glucose^2^.participant	238.333	–	–	–
cor_(Intercept).Glucose.particiant	−0.191	–	–	–
cor_(Intercept).Glucose^2^.participant	0.155	–	–	–
cor_Glucose.Glucose^2^.participant	−0.278	–	–	–
sd_Observation.Residual	124.696	–	–	–
**GCPT accuracy**				
(Intercept)	2.517	0.047	2.441	2.595
Glucose	0.476	0.622	−0.543	1.516
Glucose^2^	0.475	0.634	−0.563	1.518
sd_(Intercept).participant	0.614	–	–	–
sd_Glucose.participant	0.738	–	–	–
sd_Glucose^2^.participant	0.753	–	–	–
cor_(Intercept).Glucose.particiant	−0.028	–	–	–
cor_(Intercept).Glucose^2^.participant	−0.103	–	–	–
cor_Glucose.Glucose^2^.participant	−0.019	–	–	–
sd_Observation.Residual	0.622	–	–	–
**GCPT RT**				
(Intercept)	824.589	6.419	814.168	835.118
Glucose	−84.259	60.192	−184.337	14.849
Glucose^2^	86.000	60.621	−12.236	186.331
sd_(Intercept).participant	87.497	–	–	–
sd_Glucose.participant	151.601	–	–	–
sd_Glucose^2^.participant	132.837	–	–	–
cor_(Intercept).Glucose.particiant	−0.075	–	–	–
cor_(Intercept).Glucose^2^.participant	−0.065	–	–	–
cor_Glucose.Glucose^2^.participant	−0.356	–	–	–
sd_Observation.Residual	59.364	–	–	–

Estimates, standard errors (SE), and 95% lower and upper credible intervals (CIL, CIU) from hierarchical Bayesian models characterizing dynamic, within-person associations between glucose (centered and scaled within-person) and cognition (DSM RT, DSM accuracy, GCPT RT, GCPT accuracy) in the ≥66% EMA completion sample. Cognitive vulnerability to glucose fluctuations was operationalized with respect to quadratic terms for glucose (below: Glucose^2^).

Consistent with H2, there were meaningful individual differences in cognitive vulnerability to glucose fluctuations for RT (DSM RT, GCPT RT; refer to Supplementary Fig. 2 for visualization and Supplementary Table 2 for statistics). These results suggest that glucose fluctuations affect cognitive slowing for some individuals to a greater extent than others. Specifically, when glucose was one SD below its mean, individuals with high (+1 SD) vulnerability to glucose fluctuations responded 0.76 ms (GCPT RT) and 3.24 ms (DSM RT) slower than individuals with low (−1 SD) vulnerability to glucose fluctuations. When glucose was two SDs below its mean, individuals with high vulnerability to glucose fluctuations responded 1.8 ms (GCPT RT) and 9.30 ms (DSM RT) slower than individuals with low vulnerability to glucose fluctuations (Fig. 3b, c). We did not observe individual differences in cognitive vulnerability to glucose fluctuations for accuracy (DSM accuracy, GCPT accuracy; see Supplementary Table 2).

**Fig. 4:**
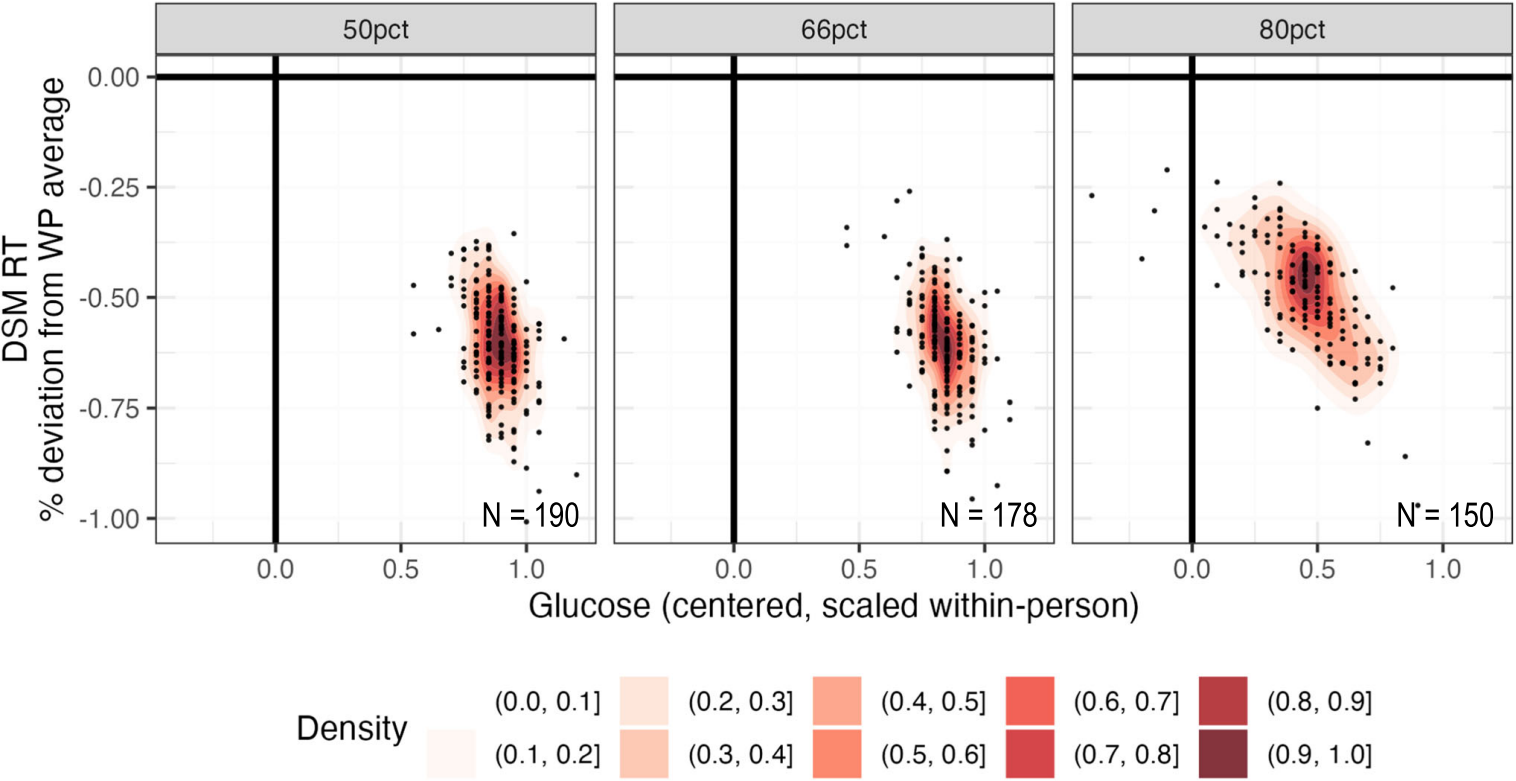
Optimal cognitive performance was associated with slightly elevated glucose levels. Optimal DSM RT (y-axis) plotted against glucose levels that were associated with optimal DSM RT (x-axis) for each EMA completion cutoff (≥50%, ≥66%, ≥80%). Along the x-axis: glucose is centered and scaled within-person (WP). Along the y-axis: optimal DSM RT is plotted as percent (%) deviation from WP average performance. More negative values indicate a larger difference between optimal and average performance. Each dot represents one participant. For most participants (red high-density regions), optimal performance occurred 0.72 standard deviations (47.49 mg/dL) above WP glucose means and was associated with 0.57% (5.30 ms) faster performance relative to WP cognitive means.

Given that DSM RT exhibited significant group (H1) and variable individual (H2) estimates of cognitive vulnerability to glucose fluctuations, we next sought to characterize optimal (i.e., fast) performance. Optimal performance consistently occurred above individuals’ glucose means, regardless of the value of those means. On average, it occurred 0.72 SDs (47.49 mg/dL) above individuals’ glucose means and was associated with 0.57% (5.30 ms) performance gain relative to individuals’ cognitive means. Glucose concentrations associated with optimal performance—and the extent to which optimal performance represented an improvement relative to typical performance—varied between individuals (Fig. 4).

### Aim 2: Clinical characteristics predicted cognitive vulnerability to glucose fluctuations

**Fig. 5:**
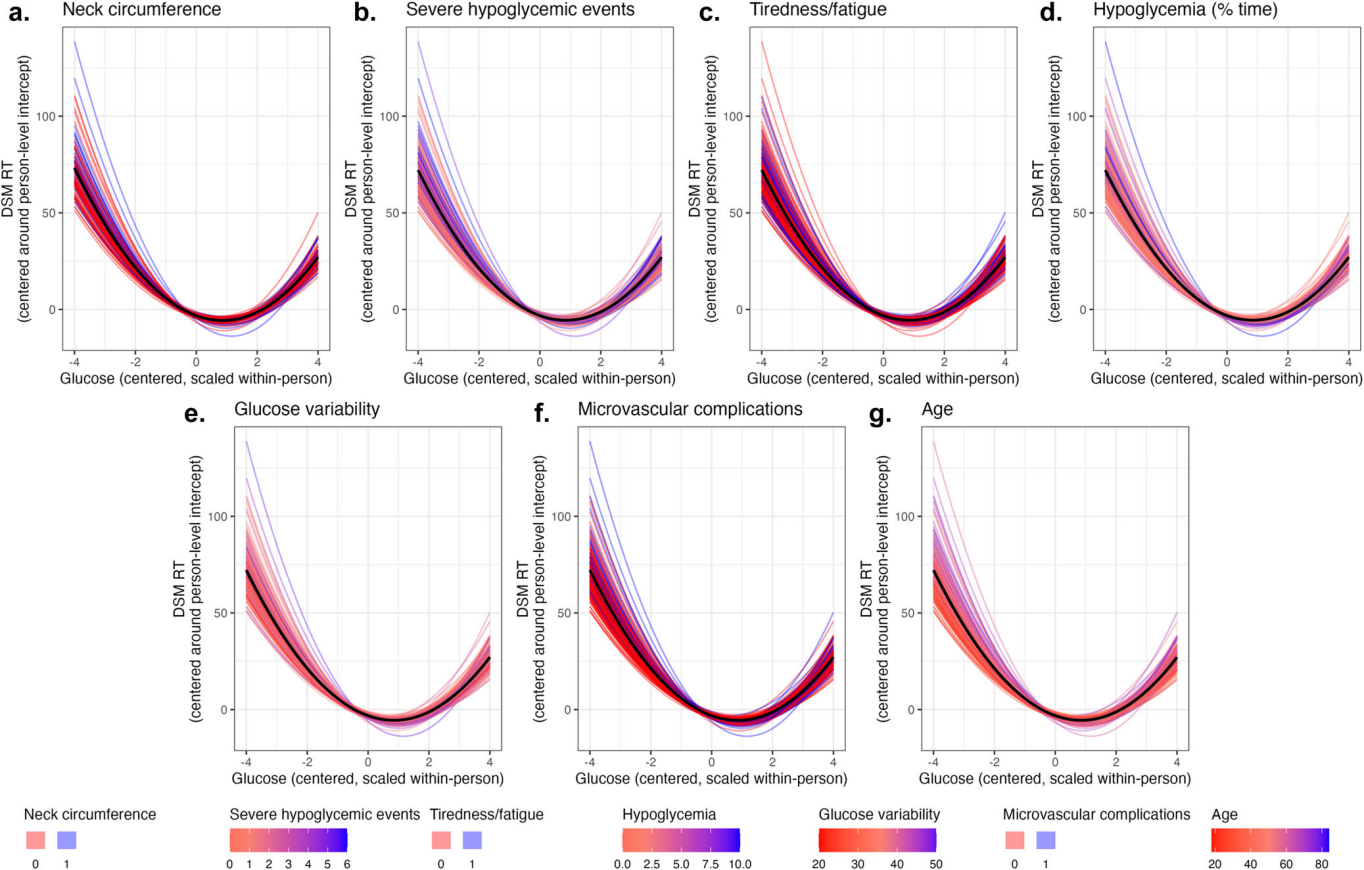
Data-driven analyses identified seven variables that explained between-person differences in cognitive vulnerability to glucose fluctuations for DSM RT. Group estimates of cognitive vulnerability to glucose fluctuations are visualized as thick black lines, and individual estimates are visualized as thin lines. The color of the individual lines reflects the value of each variable, **a**–**g**: **a** neck circumference (NeckCir_binary: [0] circumference ≤40 cm, [1] circumference > 40 cm), **b** number of lifetime severe hypoglycemic events (SevereHypoEvents: 0 [no events] to 6 [>10 events]), **c** tiredness/fatigue (tired_binary: [0] not tired during the day, [1] tired during the day), **d** percent CGM time in hypoglycemic range (gluBelow70: percent time < 70 mg/dL), **e** CGM glucose variability (gluCV: percent ratio of glucose standard deviation to glucose mean), **f** presence vs. absence of microvascular disease (microvascular_binary: [0] microvascular disease absent, [1] microvascular disease present), and **g** age (in years). Additional details about variable derivation are in Supplementary Table 3.

Data-driven lasso regression identified seven variables from a larger feature set of 58 (Supplementary Table 3) that explained individual differences in cognitive vulnerability to glucose fluctuations for DSM RT. Lasso regression tends to retain the strongest among correlated predictors (Supplementary Fig. 3)^24^, so we discuss significant results in terms of constructs rather than variables (refer to Table 4 codebook). Across EMA completion cutoffs, (1) older age, (2) greater CGM time in hypoglycemia, (3) greater number of lifetime severe hypoglycemic events, (4) presence of microvascular complication(s), (5) greater CGM glucose variability, (6) greater self-reported tiredness/fatigue, and (7) larger neck circumference predicted greater cognitive vulnerability to glucose fluctuations (Table 5, Fig. 5). To determine whether these risks specifically indicated cognitive vulnerability to glucose fluctuations, we ran additional analyses using lasso regression to predict individual differences in average cognition (Supplementary Table 4). Except for CGM glucose variability, variables that predicted individual estimates of cognitive vulnerability to glucose fluctuations also predicted individual differences in average cognition, suggesting that they represent general risk for slow responding as well as greater vulnerability to slow responding at low and high glucose.

**Table 4:** Abridged codebook

Variable	Construct	Operationalization
age	Age	Age in years at enrollment
gluBelow70	Hypoglycemia	Percent time in hypoglycemic range < 70 mg/dL
gluCV	Glucose variability	Glucose coefficient of variation
SevereHypoEvents	Severe hypoglycemic events (lifetime)	Self-reported number of lifetime episodes of low glucose requiring the assistance of another person to treat: [0] 0, [1] 1, [2] 2, [3] 3, [4] 4, [5] 5–10, [6] > 10
microvascular_binary	Microvascular disease	Presence of microvascular disease based on medical record data, i.e., retinopathy, nephropathy, neuropathy: [0] no, [1] yes
tired_binary	Tiredness/fatigue	Feeling tired and fatigued during the day, measured using the STOP-BANG questionnaire for obstructive sleep apnea^25^: [0] no, [1] yes
NeckCir_binary	Neck circumference	Neck circumference > 40 cm, measured using the STOP-BANG questionnaire for obstructive sleep apnea^25^: [0] no, [1] yes

Names, constructs, and operational definitions for person-level variables referenced in the main text.

**Table 5:** Data-driven predictors of cognitive vulnerability to glucose fluctuations

Variable	≥50%: Mean (SD, *n*)	≥66%: Mean (SD, *n*)	≥80%: Mean (SD, *n*)
mean_RMSE	34.42 (5.06, NA)	29.91 (4.33, NA)	45.86 (7.92, NA)
mean_R2	0.42 (0.01, NA)	0.35 (0.01, NA)	0.40 (0.02, NA)
**NeckCir_binary**	**0.88 (0.2, 999)**	**1.26 (0.27, 999)**	**2.12 (0.43, 1000)**
DKALast12MonthsB		0.25 (0.25, 583)	
DKANumEverB	0.62 (0.31, 674)	1.35 (0.54, 975)	
**gluBelow70**	**3.09 (0.46, 1000)**	**2.47 (0.37, 1000)**	**4.06 (1.44, 1000)**
**gluCV**	**1.93 (0.35, 1000)**	**2.16 (0.31, 1000)**	**3.08 (1.04, 1000)**
gluHours	−1.66 (0.72, 989)		−1.07 (1.25, 699)
**microvascular_binary**	**4.62 (0.47, 1000)**	**3.72 (0.34, 1000)**	**5.66 (0.84, 1000)**
NeckCir	1.4 (0.83, 728)		4.9 (2.13, 1000)
nReadings	0 (0, 840)		−0.04 (0.05, 699)
PtHypoKnowledge	1.69 (0.23, 1000)	0.57 (0.2, 990)	
**SevereHypoEvents**	**2.47 (0.48, 1000)**	**1.12 (0.24, 999)**	**6.74 (0.89, 1000)**
bloodPressure_binary	0.18 (0.08, 674)		
snoring_binary	−1.71 (0.81, 790)		
**tired_binary**	**1.39 (0.52, 790)**	**0.9 (0.46, 914)**	**1.19 (0.73, 939)**
**age**	**25.75 (0.93, 1000)**	**19.06 (0.91, 1000)**	**28.93 (1.09, 1000)**
goToSleepTypical	−0.5 (0.2, 728)	−0.67 (0.33, 914)	
hispanic_yes	0.45 (0.4, 587)		
wakeUpTypical	0.48 (0.3, 674)		

Lasso regression identified strong predictors of individual estimates of cognitive vulnerability to glucose fluctuations for DSM RT. Model performance (mean_RMSE, mean_R2) is shaded gray. Coefficient means and standard deviations (SD) were estimated over *n* = 1000 cross-validation repetitions using ≥50%, ≥66%, and ≥80% EMA completion cutoffs. N indicates the number of repetitions that retained a given predictor, and bold text indicates that a predictor was retained in over 50% of repetitions across all EMA cutoffs. Seven variables predicted increased cognitive vulnerability to glucose fluctuations across all completion cutoffs: older age (age), greater time in hypoglycemia (gluBelow70), greater number of severe hypoglycemia events (SevereHypoEvents), presence of at least one microvascular complication (microvascular_binary), greater glucose variability (gluCV), chronic fatigue (tired_binary), and larger neck circumference (NeckCir_binary).

*RMSE* cross-validated root mean squared error, *R2* cross-validated R-squared, *DKALast12MonthsB* number of diabetic ketoacidosis events in last 12 months, *DKANumEverB* number of lifetime diabetic ketoacidosis events, *gluHours* hours of glucose readings during study, *NeckCir* neck circumference in centimeters (continuous), *nReadings* number of glucose readings during study, *PtHypoKnowledge* hypoglycemia awareness (low values reflect greater awareness), bloodPressure_binary blood pressure, measured using STOP-BANG^25^, *snoring_binar* snoring, measured using STOP-BANG^25^, *goToSleepTypical* typical self-reported bedtime, *hispanic_yes* Hispanic, *wakeUpTypical* typical self-reported rise time, refer to Supplementary Table 3 for additional measurement details.

### Post-hoc analyses

Neck circumference was one of seven variables that explained individual differences in cognitive vulnerability to glucose fluctuations. Post-hoc analyses sought to clarify this unexpected finding by correlating individual estimates of cognitive vulnerability to glucose fluctuations with three additional variables (sleep apnea risk, upper body adiposity, gender) that, themselves, have been shown to correlate with neck circumference^25–28^. Correlation tests indicated that sleep apnea risk^25^ (*r* = 0.43, *p* <.001) and upper body adiposity (*r* = 0.16, *p* = 0.051) were strongly associated with individual estimates of cognitive vulnerability to glucose fluctuations. Gender (*r* = 0.11, *p* = 0.17) was not associated with individual estimates of cognitive vulnerability to glucose fluctuations.

To estimate dynamic associations between glucose and cognition independent of clinical target ranges, primary analyses used within-person glucose centering and scaling. To test the robustness of our results under different data processing conditions, we reran analyses in the ≥66% EMA completion sample using within-person glucose centering (without scaling). Consistent with Aim 1 results, we observed significant group (H1) and variable individual (H2) estimates of cognitive vulnerability to glucose fluctuations for DSM RT (statistics reported in Supplementary Tables 5, 6). Specifically, large glucose fluctuations were associated with slower and less accurate DSM performance. The rank order of individual estimates of cognitive vulnerability to glucose fluctuations was similar between primary and post hoc models (*r* [95% confidence interval] = 0.94 [0.92, 0.96]). Consistent with Aim 2 results, robust predictors from primary analyses that scaled glucose remained significant in post hoc analyses that did not scale glucose (Supplementary Table 7).

## Discussion

This study characterized dynamic, within-person associations between glucose fluctuations and cognition in adults with T1D during naturally occurring periods of hypo- (low), hyper- (high), and eu- (target) glycemia, providing foundational knowledge about when and for whom glucose fluctuations predict cognitive impairment. We hypothesized that cognitive performance would be reduced at low and high glucose, reflecting cognitive vulnerability to glucose fluctuations (H1). We also expected to observe individual differences in cognitive vulnerability to glucose fluctuations (H2). Both hypotheses were supported for processing speed (specifically, DSM RT) but not sustained attention. Next, we used data driven methods to understand whether individual differences in vulnerability to glucose fluctuations were associated with clinical features of T1D. For processing speed, we observed that greater vulnerability to glucose fluctuations was explained by (1) older age, (2) greater CGM time in hypoglycemia, (3) greater number of lifetime severe hypoglycemic events, (4) presence of microvascular complication(s), (5) greater CGM glucose variability, (6) greater self-reported tiredness/fatigue, and (7) larger neck circumference. Together, results indicate that processing speed is vulnerable to glucose fluctuations, the magnitude of this effect differs between individuals, and individual differences in vulnerability to glucose fluctuations reflect risk factors that are both specific (e.g., time in hypoglycemia) and non-specific (e.g., fatigue) to T1D.

Group estimates of cognitive vulnerability to glucose fluctuations were evident for processing speed (DSM) but not sustained attention (GCPT) and RT more so than accuracy (Supplementary Fig. 1). This pattern cannot be explained by differences in cognitive task reliability (Table 2). Consistent with present results, some researchers have posited that processing speed impairments are foundational to T1D, underlying observed impairments in other domains^29,30^. Processing speed impairments may appear stronger for speed (median RT) compared to accuracy (number correct) because speed can be measured using robust statistics, mitigating the impact of environmental disruptions unrelated to T1D^19^. Alternatively, it is possible that we did not obtain significant results for sustained attention because sustained attention is vulnerable to prolonged (e.g., hour-to-hour) rather than dynamic (moment-to-moment) changes in glucose. Pyatak et al.^23^ examined the impact of overnight glucose on next-day functioning, observing that overnight glucose variability and time in hypoglycemia prospectively predicted sustained attention. Their results suggest that sustained attention may be vulnerable to longer-term effects of glucose variability on sleep/wake regulatory processes^31^, whereas present results suggest that processing speed may be vulnerable to current glycemic status. Additional research is necessary to characterize the association between glucose and cognition across a continuum of timescales.

Given that processing speed was vulnerable to glucose fluctuations, we next sought to describe optimal processing speed, defined as the minima of quadratic curves relating glucose to reaction time. Optimal processing speed was 5.30 ms faster than average processing speed (Fig. 4). This effect size compares to effect sizes observed in studies of partial and total sleep restriction^32–35^ and suggests that most within-person variation in processing speed occurs over a relatively small range. It remains unclear whether these subtle performance gains and losses are perceived by individuals and/or have practical implications in T1D (e.g., for driving).

Optimal processing speed occurred at glucose concentrations slightly above participants’ glucose means, regardless of the absolute level (e.g., euglycemic vs. hyperglycemic) of those means. Metabolic habituation may explain why optimal performance occurred near participants’ means. Evidence for habituation comes from previous studies demonstrating that repeated exposure to hypoglycemia impairs subjective awareness of hypoglycemia^36,37^, and sustained exposure to hyperglycemia triggers autonomic responses to hypoglycemia at higher thresholds (e.g., 110 mg/dL rather than 70 mg/dL)^38^. Our results suggest that individuals may habituate to their typical glucose range and thereafter perform cognitively optimally within that range. The opposite is also possible: individuals with T1D may gravitate toward glucose ranges that support optimal cognitive performance. Future studies are required to disentangle these possibilities. Meal timing may further explain why optimal performance occurred slightly above (rather than at) participants’ glucose means. Glucose and insulin are elevated after meals, and insulin is cognitively enhancing^39^. Longitudinal burst studies^40,41^ are necessary to evaluate the cognitive effects of gradual (vs. rapid) increases (vs. decreases) in average glucose. This work may inform recommendations to improve diabetes self-management without compromising cognition.

Although processing speed was generally vulnerable to glucose fluctuations, some individuals were more vulnerable to glucose fluctuations than others (Fig. 3b, c). Individuals who were more vulnerable to glucose fluctuations tended to exhibit poorer diabetes control, including greater CGM time in hypoglycemia, greater number of lifetime severe hypoglycemic events, greater CGM glucose variability, and microvascular complication(s) (Table 5, Fig. 5). In addition to diabetes-specific risks, we observed greater vulnerability to glucose fluctuations among older individuals, individuals with more self-reported fatigue, and individuals with larger neck circumference. The latter result was unexpected and may reflect the fact that neck circumference correlates with several well-established risk factors for cognitive impairment, including sleep apnea risk and upper body adiposity^42–47^. Although we included sleep apnea risk and upper body adiposity in data-driven models, our approach (lasso regression) tends to select only the strongest among correlated features^24^. Together, results of data-driven analyses identify several diabetes-specific (e.g., time in hypoglycemia) and non-specific (e.g., neck circumference, sleep apnea, upper body adiposity, fatigue) risks that indicate increased cognitive vulnerability to glucose fluctuations. Individuals with these risk factors may be advised to limit consequential, speed-dependent cognitive tasks in moments when glucose is considerably above or below its typical level.

We found that a similar profile of diabetes-specific and non-specific risks also predicted individual differences in average processing speed (Supplementary Table 4), indicating overlap between risks for slow processing speed and risks for cognitive vulnerability to glucose fluctuations. These results replicate prior research implicating suboptimal diabetes control in cognitive impairment^23,25,48–51^, and they provide empirical support for theories positing that accumulated diabetes-related insults increase cognitive vulnerability to glucose fluctuations^52–54^. It follows that cognitive vulnerability to glucose fluctuations may be a sensitive digital biomarker (i.e., digital marker of normal or pathogenic biological processes)^55^ of neurocognitive dysfunction in T1D. Future evidence that cognitive vulnerability predicts long-term clinical outcomes would suggest the utility of digital cognitive assessments for remote risk screening.

This work clarifies the natural time course of dynamic, within-person associations between glucose and cognition in T1D. There are, however, limitations to consider. First, we required 24-hour access to a smartphone with reliable internet access. This requirement may have biased sampling towards individuals with higher socioeconomic status. Although “bring your own device” (BYOD) studies increase accessibility and scalability^20,56,57^, future studies might mitigate sampling biases by providing participants with smartphones. Second, the present sample was predominantly white and non-Hispanic, limiting generalizability to other racial and ethnic groups. Third, CGM became standard of care during data collection. Thereafter, we allowed participants (*n* = 130) to use their personal, clinically prescribed (unblinded) CGM in addition to study administered (blinded) CGM. Notably, blinding did not account for individual differences in cognitive vulnerability to glucose fluctuations (Table 5). Finally, age was highly correlated with mean DSM RT (*r* [95% confidence interval] = 0.67 [0.59, 0.74]). This suggests that the effects of age on cognitive vulnerability to glucose fluctuations may reflect, in part, between-person differences in mean performance. Future efforts to develop age-based norms for cognitive EMA will help to address this limitation.

The present study leveraged recent advances in CGM and cognitive EMA to characterize dynamic, within-person associations between glucose and cognition in naturalistic environments. Results demonstrate that processing speed is vulnerable to glucose fluctuations, the magnitude of this effect differs between individuals, and individual differences in vulnerability to glucose fluctuations reflect diabetes-specific (e.g., time in hypoglycemia) as well as non-specific (e.g., fatigue) risks. These findings have implications for risk screening and behavioral intervention in T1D. With respect to risk screening, our results suggest that individual estimates of cognitive vulnerability to glucose fluctuations can be feasibly, remotely ascertained using scalable digital technologies and show preliminary validity as digital biomarkers of neurocognitive dysfunction in T1D. Future research is required to evaluate the long-term temporal stability and clinical utility of individual estimates of cognitive vulnerability to glucose fluctuations. With respect to intervention, our results focus attention on a limited number of modifiable risks that may be targeted to improve cognitive performance and reduce cognitive vulnerability to glucose fluctuations. Randomized control trials are necessary to lend empirical support to interventions targeting these risks. In the present study, T1D provided a powerful model for understanding the pathophysiology of cognitive fluctuations in a clinical population that experiences frequent, short-term cognitive impairment^4,8,9^. Beyond T1D, this work demonstrates how data from physiological sensors and EMA can be integrated to better understand individual differences in daily functioning and clinical risk.

## Methods

### Participants

Adults with T1D (*N* = 200, 107 female) were recruited from diabetes and endocrinology centers at Mayo Clinic in Rochester, Minnesota, State University of New York (SUNY) Upstate Medical University, University of Pennsylvania, and Advent Health in Orlando, Florida (Table 1). To enroll, participants were required to be over 18 years old, diagnosed with T1D for >1 year, and fluent in English. They were also required to have 24-hour access to a personal smartphone with a reliable internet connection, demonstrate understanding of the EMA protocol, and agree to comply with it. Participants were excluded based on the following: inability to complete cognitive assessments owing to significant visual, motor, hearing, or cognitive impairment; any medical or psychiatric condition or treatment that was determined by the principal investigators to interfere with completion of the study; and inability to complete EMAs (scheduled 9:00 AM–9:00 PM) due to night shift work, planned travel across time zones, and/or other circumstances that would systematically interfere with the ability to complete assessments. Written informed consent was obtained prior to enrollment, and the study procedures were approved by the Jaeb Center for Health Research IRB. The Jaeb Center served as the clinical coordinating center.

Participants completed initial clinic visits (~2 hours) and baseline cognitive data collection (~45 minutes) prior to EMA. Most clinic visits were completed in person; however, a subset of participants (*n* = 25) completed clinic visits virtually due to the COVID-19 pandemic. Baseline cognitive data collection was completed virtually. Participants were excluded if they completed <50% of EMAs. This was a pre-specified criterion based on concern that low EMA compliance would introduce sampling bias (e.g., increased sampling during periods of euglycemia relative to hypoglycemia). To encourage compliance, participants received bonus compensation if they completed >80% of EMAs.

### Cognitive tasks

We administered three cognitive tasks during EMA: digit symbol matching (DSM), gradual onset continuous performance test (GCPT), and multiple object tracking (MOT). Tasks were selected based on (1) their ability to measure performance in domains that are sensitive to cognitive impairment and/or change in T1D^30,58–60^ and (2) mobile pilot testing demonstrating high completion, high usability (e.g., minimal participant-reported burden), minimal range restriction, and good between-person reliability^61^. In the present manuscript, we did not analyze MOT data due to low within-person reliability <0.3^19^, which limits variance that may be explained by time-varying predictors such as glucose^62^. Refer to^63,64^ for MOT task details. Links to tasks, including task instructions and practice trials, are available on GitHub: https://github.com/zwihawks/MomentaryCogT1D/blob/main/CogEMA_tasks.rtf.

TestMyBrain DSM for EMA (Fig. 2a) is a visuospatial task with demands on processing speed and short-term working memory^65^. It was adapted for remote administration from the Wechsler Adult Intelligence Scale digit symbol coding task^66,67^ and has been previously validated for cognitive EMA in T1D^68^. During administration, participants were presented with both a target and a digit-symbol pairing key (Fig. 2a). Participants were instructed to press (using their device’s touchscreen) the digit that was paired with the target symbol in the key. There was no response deadline. After response selection, the next target symbol appeared. In each EMA session, six symbols were sampled from a larger set of thirty. Symbols were paired with digits in a 2:1 symbol-to-digit ratio. Digit-symbol pairings varied across, but not within, EMA sessions. Accuracy was recorded as the number of correct responses in 30 seconds. Reaction time (RT) was recorded as the median RT for correct responses.

TestMyBrain GCPT for EMA (Fig. 2b) is an executive functioning task that requires sustained attention, cognitive control, and response inhibition^69,70^. It has been previously validated for cognitive EMA in T1D^68^. In each trial, participants viewed a circular, grayscale image of a city or mountain (Fig. 2b). They were instructed to respond (by pressing their device’s touchscreen) when the image depicted a city (80% of trials), and they were instructed to withhold a response when the image depicted a mountain (20% of trials). GCPT for EMA consisted of seventy-five 800 millisecond trials (test duration = 60 seconds). Images faded in and out between trials. Accuracy was recorded as discrimination sensitivity (d-prime)^71^, and RT was recorded as the median RT for correct responses.

### Ecological Momentary Assessment (EMA)

Participants received text messages prompting them to complete brief (~7-minute) EMA sessions. Each EMA session included self-report questionnaires and cognitive assessments. Text messages (3 per day x 15 days = 45 total) were delivered at random times within 4-hour windows: morning (9:00 AM–12:59 PM, according to the local time zone), afternoon (1:00–4:59 PM), and evening (5:00–9:00 PM). Upon receiving a text, participants had 30 minutes to start an EMA session. If needed, they received a text reminder after 25 minutes had elapsed. Self-report questionnaires were not analyzed. Refer to ref. 61 for questionnaire details.

Cognitive test batteries were identical within EMA sessions (e.g., all participants completed the same test versions in the same order at time 1) but varied across sessions (e.g., each participant completed different test versions at time 1 vs. time 2, and tests were presented in different orders at time 1 vs. time 2). Varying the test versions across sessions discouraged participants from relying on memory, ensuring that tasks remained valid measures of performance within the intended cognitive domains. As part of onboarding, participants reviewed task instructions, completed practice trials, and received corrective feedback.

### Continuous Glucose Monitoring (CGM)

Study-administered CGM (Dexcom G6) devices were inserted during the initial clinic visit and worn for a maximum of twenty days. After ten days, participants were instructed to replace the original study administered CGM device with a second one^72^. Study-administered CGM devices did not require manual calibration, and participants were blinded to their readings. Nonetheless, use of CGM is part of standard care in T1D^73^, and some participants (*n* = 128) utilized personal CGM devices in addition to study-administered blinded CGM devices. These participants had access to measurements from personal CGM devices throughout the study.

### Data cleaning and processing

Analyses were performed in R v4.1.1^74^ using the *tidyverse* package for data exclusion and visualization^75^, the *rstanarm* and *tidybayes* packages for hypothesis-driven (hierarchical Bayesian) modeling^76,77^, and the *glmnet* package for data-driven (lasso) modeling^78,79^.

Participants were required to provide >72 hours of raw data from study administered CGM devices. Two participants (1 female) did not provide sufficient CGM data and were excluded from analyses. One participant (female) was excluded due to a protocol deviation (two study-administered CGM devices were worn simultaneously). Within participants, consistent with manufacturer’s instructions, we excluded the first 24 hours data from each study-administered device due to reduced accuracy^80^. Exclusions occurred prior to EMA for the first device (inserted during the clinic visit) and around day nine of EMA for the second device (inserted mid-study to replace the first device after 10 days of wear). To characterize group estimates of cognitive vulnerability to glucose fluctuations (Aim 1), CGM and EMA data were time-aligned by subsampling CGM observations from 0–5 minutes prior to EMA. To examine individual differences in cognitive vulnerability to glucose fluctuations (Aim 2), person-level indicators of glycemic control (e.g., average glucose, glucose coefficient of variation, hypo- and hyperglycemic event rates, percent time in hypo- and hyperglycemia) were computed from CGM timeseries as previously described^81–84^. Variable distributions for Aims 1-2 are provided in Supplementary Figs. 4,5, and codebooks for Aims 1-2 are provided in Supplementary Table 3.

EMA data were excluded when task performance was comparable to chance or unlikely to represent adequate and expected effort, based on the following criteria: DSM accuracy less than 50%, DSM number correct less than six, and GCPT omission errors greater than 50%^56^. To ensure consistency across participants and sessions, we required responses to be registered using touchscreens, and we required tasks to be marked as complete (e.g., browser did not close prematurely)^19^. Five participants (all male) were excluded because they did not meet these quality control criteria in 50% or more of possible EMA sessions. Two participants (1 female) were excluded due to a technical anomaly that allowed them to complete more than 45 EMA sessions.

### Aim 1 analysis: Characterizing dynamic, within-person associations between glucose and cognition

We used hierarchical Bayesian modeling to obtain group (aggregated across all participants in the sample) and individual (specific to each participant) estimates of dynamic, within-person associations between glucose and cognition. Estimation used Gaussian family response distributions and weakly informative (default) priors^76^. Consistent with laboratory studies^8–15^, we hypothesized (H1) that cognitive performance would be reduced at low and high glucose, reflecting cognitive vulnerability to glucose fluctuations. We also hypothesized (H2) that individuals would differ in their cognitive vulnerability to glucose fluctuations.

Target glucose ranges in people with T1D are based on group data indicating risk for acute and long-term diabetes complications, and it is unclear whether boundaries defining these ranges function similarly in terms of short-term cognitive performance^38,85,86^. To estimate dynamic associations between glucose and cognition independent of clinical target ranges, we used within-person centering and scaling. Specifically, we centered glucose around person-level means and scaled glucose relative to person-level standard deviations. As a test of robustness, we ran post-hoc analyses using within-person centering but not scaling.

To capture cognitive vulnerability to low and high glucose fluctuations, we modeled glucose using orthogonal quadratic polynomials. Orthogonalizing polynomials allowed us to eliminate collinearity between predictors and assess the independent contributions of linear and quadratic terms in explaining outcome variation^87,88^:

1 $$\begin{array}{l}{Y}_{{ij}}={\gamma }_{00}+{u}_{0j}+{\gamma }_{10}* {{glucose}}_{{ij}}+{u}_{1j}* {{glucose}}_{{ij}}\\\qquad+\,{\gamma }_{20}* {{glucose}}_{{ij}}^{2}+{u}_{2j}* {{glucose}}_{{ij}}^{2}+{\varepsilon }_{{ij}}\end{array}$$

${Y}_{{ij}}$ represents cognitive score *Y* in EMA *i* for participant *j*, intercepts (group: γ_00_; individual: *u*_0*j*_) represent average cognitive performance at average glucose, linear terms for glucose (group: γ_10_; individual deviation: *u*_1*j*_) represent the strength of linear associations between glucose and cognition at average glucose, and quadratic terms for glucose (group: γ_20_; individual deviation: *u*_2*j*_) represent curvilinear (U-shaped) associations between glucose and cognition, capturing cognitive vulnerability to glucose fluctuations. To eliminate divergent transitions and obtain recommended effective sample sizes^89^, outlying observations greater than three standard deviations from the mean of glucose^2^ were excluded prior to modeling, and parameters (target average acceptance probability, number of iterations) were manually tuned. Final models converged with potential scale reduction factor ${\widehat{\left(\right.R}\left.\right)}\le 1.01$^89,90^.

Significance was evaluated using 95% credible intervals (CIs). Significant group estimates for glucose^2^ (γ_20_) provided support for H1. Significantly variable individual estimates for glucose^2^ (*u*_2*j*_) provided support for H2. Variation was operationalized with respect to the tau matrix and quantified using the test for practical equivalence with region bounds $\left[0,0.2* {{SD}}_{y}\right]$^91,92^. Optimal cognitive performance was estimated as the minima (for RT) or maxima (for accuracy) of quadratic curves relating glucose to cognition. It was interpreted with respect to percent deviation from typical cognition (i.e., group and individual intercepts). To localize optimal performance, we predicted cognitive performance over the domain of glucose (centered and scaled: [−4, 4], step size = 0.05) by averaging draws (*n* = 1,000) from the expectation of the posterior predictive distribution^77^. Summary statistics are reported as weighted averages across EMA completion samples, and predictions were centered around participant-level intercepts for visualization. Additional model formulas and R syntax are provided in Supplementary Note 1.

To evaluate the impact of observation vs. sample selection biases, models were run for each cognitive outcome (DSM RT and accuracy, GCPT RT and accuracy) across three EMA completion cutoffs (≥50%, ≥66%, ≥80%). With low (e.g., ≥50%) completion, we risk sampling bias: EMA observations may be systematically missing when glucose is very low or very high. With high (e.g., ≥80%) completion, we risk selection bias: individuals excluded from analysis may differ systematically from those included in analysis. We only interpreted results that were significant across all completion cutoffs.

### Aim 2 analysis: Examining individual differences in cognitive vulnerability to glucose fluctuations

We used data-driven lasso regression to identify participant characteristics that predicted individual differences in cognitive vulnerability to glucose fluctuations. Given dependencies between aims, Aim 2 analyses focused on cognitive outcomes for which Aim 1 hypotheses were supported. In lasso regression, strong predictors were selected from a larger feature space (*n* = 58 variables, described in Supplementary Table 3) that included standardized clinical, CGM, and demographic variables relevant to diabetes self-management. Hyperparameter tuning was performed using *cv.glmnet* with 10-fold cross-validation. Coefficients were estimated by refitting tuned models to the full dataset. This process was repeated 1,000 times using each EMA completion cutoff (≥50%, ≥66%, ≥80%). Predictors were considered robust if they were selected in over 50% of repetitions for all completion cutoffs.

### Reporting summary

Further information on research design is available in the Nature Research Reporting Summary linked to this article.

### Supplementary information

Supplemental Material

Reporting Summary

## Data Availability

Data for the current study are available from the corresponding author upon reasonable request.

## Code availability

Code to analyze and visualize data is available on GitHub, https://github.com/zwihawks/MomentaryCogT1D.
