# Supplementary material for: Dynamic associations between glucose and ecological momentary cognition in Type 1 Diabetes
Source: NPJ Digit Med. 2024 Mar 18;7:59. doi: 10.1038/s41746-024-01036-5 (PMC10948782; doi:10.1038/s41746-024-01036-5)
Supplement: Supplementary file 1 — Supplemental Material [file 41746_2024_1036_MOESM1_ESM.pdf]

Supplement

Tables

Supplementary Table 1……………………………………………………… page 2
Supplementary Table 2……………………………………………………… page 6
Supplementary Table 3………..…………………………………………… page 7
Supplementary Table 4……………………………………………………… page 9
Supplementary Table 5 ……………………………………………………... page 10
Supplementary Table 6……………………………………………………… page 11
Supplementary Table 7……………………………………………………… page 12

Figures

Supplementary Figure 1 ……………………………………………………. page 13
Supplementary Figure 2 ……………………………………………………. page 14
Supplementary Figure 3 ……………………………………………………. page 15
Supplementary Figure 4………..……………………………………………page 16
Supplementary Figure 5………..……………………………………………page 17

Notes

Supplementary Note 1 ……………………………………………………… page 18

Supplementary Table 1. Aim 1, H1: Estimates, standard errors (std.error), and 95% credible intervals (conf.low, conf.high) from hierarchical Bayesian models examining associations between glucose (centered and scaled within-person) and cognitive performance (DSM RT, DSM accuracy, GCPT RT, GCPT accuracy) across three EMA completion cut-offs (50, 66, 80%). Cognitive vulnerability to glucose fluctuations was operationalized with respect to quadratic terms for glucose (Glucose^2).

term	estimate	std.error	conf.low	conf.high	group
**50pct_dsm_accuracy**					
(Intercept)	21.481	0.271	21.022	21.929	NA
Glucose	10.060	2.636	5.750	14.428	NA
Glucose^2	-3.865	2.649	-8.225	0.405	NA
sd_(Intercept).user_id	3.778	NA	NA	NA	user_id
sd_Glucose. user_id	3.871	NA	NA	NA	user_id
sd_Glucose^2. user_id	3.725	NA	NA	NA	user_id
cor_(Intercept).Glucose.user_id	0.283	NA	NA	NA	user_id
cor_(Intercept).Glucose^2.user_id	0.162	NA	NA	NA	user_id
cor_Glucose.Glucose^2.user_id	-0.050	NA	NA	NA	user_id
sd_Observation.Residual	2.594	NA	NA	NA	Residual
**50pct_dsm_RT**					
(Intercept)	955.471	15.899	928.795	981.477	NA
Glucose	-378.476	127.253	-590.666	-165.798	NA
Glucose^2	254.794	127.939	44.520	467.420	NA
sd_(Intercept).user_id	221.078	NA	NA	NA	user_id
sd_Glucose. user_id	305.499	NA	NA	NA	user_id
sd_Glucose^2. user_id	236.957	NA	NA	NA	user_id
cor_(Intercept).Glucose.user_id	-0.200	NA	NA	NA	user_id
cor_(Intercept).Glucose^2.user_id	0.202	NA	NA	NA	user_id
cor_Glucose.Glucose^2.user_id	-0.285	NA	NA	NA	user_id
sd_Observation.Residual	125.166	NA	NA	NA	Residual
**50pct_gcpt_accuracy**					
(Intercept)	2.471	0.048	2.393	2.552	NA
Glucose	0.324	0.643	-0.726	1.370	NA
Glucose^2	0.384	0.645	-0.663	1.445	NA
sd_(Intercept).user_id	0.644	NA	NA	NA	user_id
sd_Glucose. user_id	0.718	NA	NA	NA	user_id
sd_Glucose^2. user_id	0.741	NA	NA	NA	user_id
cor_(Intercept).Glucose.user_id	0.015	NA	NA	NA	user_id
cor_(Intercept).Glucose^2.user_id	-0.043	NA	NA	NA	user_id
cor_Glucose.Glucose^2.user_id	-0.035	NA	NA	NA	user_id
sd_Observation.Residual	0.627	NA	NA	NA	Residual
**50pct_gcpt_RT**					
(Intercept)	822.781	6.394	812.157	833.200	NA
Glucose	-96.166	61.284	-195.874	4.337	NA
Glucose^2	68.954	60.628	-31.078	171.108	NA

term	estimate	std.error	conf.low	conf.high	group
sd_(Intercept).user_id	86.806	NA	NA	NA	user_id
sd_Glucose. user_id	148.232	NA	NA	NA	user_id
sd_Glucose^2. user_id	128.691	NA	NA	NA	user_id
cor_(Intercept).Glucose.user_id	-0.024	NA	NA	NA	user_id
cor_(Intercept).Glucose^2.user_id	-0.009	NA	NA	NA	user_id
cor_Glucose.Glucose^2.user_id	-0.295	NA	NA	NA	user_id
sd_Observation.Residual	60.553	NA	NA	NA	Residual
**66pct_dsm_accuracy**					
(Intercept)	21.514	0.283	21.056	21.993	NA
Glucose	9.786	2.585	5.521	14.049	NA
Glucose^2	-4.406	2.642	-8.701	-0.024	NA
sd_(Intercept).user_id	3.804	NA	NA	NA	user_id
sd_Glucose. user_id	3.862	NA	NA	NA	user_id
sd_Glucose^2. user_id	3.695	NA	NA	NA	user_id
cor_(Intercept).Glucose.user_id	0.297	NA	NA	NA	user_id
cor_(Intercept).Glucose^2.user_id	0.119	NA	NA	NA	user_id
cor_Glucose.Glucose^2.user_id	-0.058	NA	NA	NA	user_id
sd_Observation.Residual	2.575	NA	NA	NA	Residual
**66pct_dsm_RT**					
(Intercept)	955.155	16.433	927.862	981.908	NA
Glucose	-373.036	128.264	-582.097	-164.594	NA
Glucose^2	261.336	127.639	51.143	468.944	NA
sd_(Intercept).user_id	224.063	NA	NA	NA	user_id
sd_Glucose. user_id	312.381	NA	NA	NA	user_id
sd_Glucose^2. user_id	238.333	NA	NA	NA	user_id
cor_(Intercept).Glucose.user_id	-0.191	NA	NA	NA	user_id
cor_(Intercept).Glucose^2.user_id	0.155	NA	NA	NA	user_id
cor_Glucose.Glucose^2.user_id	-0.278	NA	NA	NA	user_id
sd_Observation.Residual	124.696	NA	NA	NA	Residual
**66pct_gcpt_accuracy**					
(Intercept)	2.517	0.047	2.441	2.595	NA
Glucose	0.476	0.622	-0.543	1.516	NA
Glucose^2	0.475	0.634	-0.563	1.518	NA
sd_(Intercept).user_id	0.614	NA	NA	NA	user_id
sd_Glucose. user_id	0.738	NA	NA	NA	user_id
sd_Glucose^2. user_id	0.753	NA	NA	NA	user_id
cor_(Intercept).Glucose.user_id	-0.028	NA	NA	NA	user_id
cor_(Intercept).Glucose^2.user_id	-0.103	NA	NA	NA	user_id
cor_Glucose.Glucose^2.user_id	-0.019	NA	NA	NA	user_id
sd_Observation.Residual	0.622	NA	NA	NA	Residual
**66pct_gcpt_RT**					

term	estimate	std.error	conf.low	conf.high	group
(Intercept)	824.589	6.419	814.168	835.118	NA
Glucose	-84.259	60.192	-184.337	14.849	NA
$Glucose^2$	86.000	60.621	-12.236	186.331	NA
sd_(Intercept).user_id	87.497	NA	NA	NA	user_id
sd_Glucose. user_id	151.601	NA	NA	NA	user_id
sd_$Glucose^2$. user_id	132.837	NA	NA	NA	user_id
cor_(Intercept).Glucose.user_id	-0.075	NA	NA	NA	user_id
cor_(Intercept).$Glucose^2$.user_id	-0.065	NA	NA	NA	user_id
cor_Glucose.$Glucose^2$.user_id	-0.356	NA	NA	NA	user_id
sd_Observation.Residual	59.364	NA	NA	NA	Residual
**80pct_dsm_accuracy**					
(Intercept)	21.815	0.307	21.305	22.318	NA
Glucose	7.174	2.575	2.945	11.430	NA
$Glucose^2$	-3.674	2.547	-7.902	0.584	NA
sd_(Intercept).user_id	3.714	NA	NA	NA	user_id
sd_Glucose. user_id	4.036	NA	NA	NA	user_id
sd_$Glucose^2$. user_id	3.756	NA	NA	NA	user_id
cor_(Intercept).Glucose.user_id	0.335	NA	NA	NA	user_id
cor_(Intercept).$Glucose^2$.user_id	0.140	NA	NA	NA	user_id
cor_Glucose.$Glucose^2$.user_id	-0.062	NA	NA	NA	user_id
sd_Observation.Residual	2.536	NA	NA	NA	Residual
**80pct_dsm_RT**					
(Intercept)	939.441	17.940	910.713	969.615	NA
Glucose	-201.428	123.217	-402.290	0.239	NA
$Glucose^2$	284.118	121.556	86.433	483.379	NA
sd_(Intercept).user_id	215.811	NA	NA	NA	user_id
sd_Glucose. user_id	434.094	NA	NA	NA	user_id
sd_$Glucose^2$. user_id	273.457	NA	NA	NA	user_id
cor_(Intercept).Glucose.user_id	-0.025	NA	NA	NA	user_id
cor_(Intercept).$Glucose^2$.user_id	0.231	NA	NA	NA	user_id
cor_Glucose.$Glucose^2$.user_id	-0.252	NA	NA	NA	user_id
sd_Observation.Residual	116.762	NA	NA	NA	Residual
**80pct_gcpt_accuracy**					
(Intercept)	2.540	0.052	2.453	2.627	NA
Glucose	0.147	0.616	-0.875	1.162	NA
$Glucose^2$	0.682	0.633	-0.341	1.709	NA
sd_(Intercept).user_id	0.634	NA	NA	NA	user_id
sd_Glucose. user_id	0.787	NA	NA	NA	user_id
sd_$Glucose^2$. user_id	0.800	NA	NA	NA	user_id
cor_(Intercept).Glucose.user_id	0.079	NA	NA	NA	user_id
cor_(Intercept).$Glucose^2$.user_id	-0.172	NA	NA	NA	user_id

term	estimate	std.error	conf.low	conf.high	group
cor_Glucose.Glucose^2.user_id	-0.011	NA	NA	NA	user_id
sd_Observation.Residual	0.610	NA	NA	NA	Residual
**80pct_gcpt_RT**					
(Intercept)	822.693	7.586	810.434	835.683	NA
Glucose	-91.296	61.953	-193.707	11.735	NA
Glucose^2	92.467	61.617	-9.335	194.856	NA
sd_(Intercept).user_id	89.615	NA	NA	NA	user_id
sd_Glucose. user_id	147.743	NA	NA	NA	user_id
sd_Glucose^2. user_id	132.530	NA	NA	NA	user_id
cor_(Intercept).Glucose.user_id	-0.054	NA	NA	NA	user_id
cor_(Intercept).Glucose^2.user_id	-0.007	NA	NA	NA	user_id
cor_Glucose.Glucose^2.user_id	-0.310	NA	NA	NA	user_id
sd_Observation.Residual	59.674	NA	NA	NA	Residual

Supplementary Table 2. Aim 1, H2: Variation in individual estimates of cognitive vulnerability to glucose fluctuations (CV-GF), evaluated using the test of practical equivalence. Variation was considered significant and marginally significant when high density intervals (HDI; [HDI_low, HDI_high]) and regions of practical equivalence (ROPE) did not overlap (% inside ROPE = 0.000) with credible intervals (CIs) = 0.95 and 0.90, respectively. DSM RT and GCPT RT exhibited significant variation in individual estimates of CV-GF across all EMA completion cut-offs.

EMA cut-off	Test	Outcome	CI	% inside ROPE	HDI_low	HDI_high
50%	DSM	Accuracy	0.90	0.000	1.207	43.742
50%	DSM	Accuracy	0.95	0.008	0.760	56.408
50%	DSM	RT	0.90	0.000	4598.483	183535.243
50%	DSM	RT	0.95	0.000	2977.893	237801.065
50%	GCPT	Accuracy	0.90	0.293	0.039	1.847
50%	GCPT	Accuracy	0.95	0.304	0.025	2.515
50%	GCPT	RT	0.90	0.000	809.730	66341.048
50%	GCPT	RT	0.95	0.000	516.830	87559.258
66%	DSM	Accuracy	0.90	0.000	1.235	42.567
66%	DSM	Accuracy	0.95	0.006	0.798	55.293
66%	DSM	RT	0.90	0.000	4569.363	185852.757
66%	DSM	RT	0.95	0.000	2921.443	244986.612
66%	GCPT	Accuracy	0.90	0.290	0.038	1.964
66%	GCPT	Accuracy	0.95	0.301	0.025	2.710
66%	GCPT	RT	0.90	0.000	876.881	68912.633
66%	GCPT	RT	0.95	0.000	573.939	89038.734
80%	DSM	Accuracy	0.90	0.000	1.303	42.761
80%	DSM	Accuracy	0.95	0.003	0.829	55.172
80%	DSM	RT	0.90	0.000	5029.969	235783.083
80%	DSM	RT	0.95	0.000	3138.364	292176.040
80%	GCPT	Accuracy	0.90	0.259	0.043	2.245
80%	GCPT	Accuracy	0.95	0.272	0.026	3.044
80%	GCPT	RT	0.90	0.000	894.806	66984.339
80%	GCPT	RT	0.95	0.000	564.299	88411.152

Supplementary Table 3. Codebook.

Aim 1.

Variable	Construct	Measurement	Operationalization
cgm	Glucose	Continuous glucose monitor	mg/dL, sampled every five minutes
dsm: medianRTc	Processing speed (reaction time)	Digit symbol matching	Median reaction time on correct responses
dsm: num_correct	Processing speed (accuracy)	Digit symbol matching	Number of correct responses
gcpt: dprime	Sustained attention (accuracy)	Gradual onset continuous performance test	z(Hit rate) - z(False Alarm rate)
gcpt: medianRTc	Sustained attention (reaction time)	Gradual onset continuous performance test	Median reaction time on correct responses to targets

Aim 2.

Variable	Construct	Operationalization
age	Age	Age in years at enrollment
wakeUpTypical	Rise time	Typical week, self-reported typical rise [0-24]
wakeUpEarliest	Rise time	Typical week, self-reported earliest rise [0-24]
wakeUpLatest	Rise time	Typical week, self-reported latest rise [0-24]
goToSleepTypical	Bedtime	Typical week, self-reported typical bedtime [0-24]
goToSleepEarliest	Bedtime	Typical week, self-reported earliest bedtime [0-24]
goToSleepLatest	Bedtime	Typical week, self-reported latest bedtime [0-24]
education_num	Education	[0] high school, [1] technical or some college, [2] college, [3] masters, [4] graduate degree (e.g., PhD, JD, MD)
ethnicity_africanOrBlack	Race	African or Black: [0] no, [1] yes
ethnicity_europeanOrWhite	Race	European or White: [0] no, [1] yes
hispanic_yes	Ethnicity	Hispanic: [0] no, [1] yes
HbA1cTestRes	Average glucose (~3 months)	HbA1c results
Weight	Weight	Kilograms (kg), measured during in-person clinic visit
Height	Height	Centimeters (cm), measured during in-person clinic visit
BldPrSys	Blood pressure	Blood pressure systolic, measured during in-person clinic visit
BldPrDia	Blood pressure	Blood pressure diastolic, measured during in-person clinic visit
PEHeartRt	Heart rate	Heart rate (beats per minute), measured during in-person clinic visit
WaistCir	Waist circumference	Centimeters (cm), measured during in-person clinic visit or extracted from medical record
NeckCir	Neck circumference	Centimeters (cm), measured during in-person clinic visit or extracted from medical record
DiagAge	Age at diagnosis	Age in years at T1D diagnosis
UnitsInsTotal	Insulin	Total daily insulin in units
UnitsInsBasalOrLongAct	Insulin	Total daily basal or long acting insulin units
PtHypoKnowledge	Hypoglycemic awareness	How does participant rate their knowledge of when hypoglycemic states are commencing? [1] always aware through [7] never aware
gluMean	Average glucose	Mean glucose during study
gluLBGI	Low glucose	Low blood glucose index during study
gluHBGI	High glucose	High blood glucose index during study
gluHours	Data quality	Hours of glucose readings during study
nReadings	Data quality	Number of glucose readings during study
gluSD	Glucose variability	Standard deviation of glucose during study
gluMin	Low glucose	Minimum glucose during study
gluMax	High glucose	Maximum glucose during study
gluMAGE	Glucose variability	Mean amplitude of glycemic excursions during study
gluHyper300Rate	Hyperglycemia	Hyperglycemic (≥ 300 mg/dL) event rate per week during study
gluHypo54Rate	Hypoglycemia	Hypoglycemic (≤ 54 mg/dL) event rate per week during study
gluInRange	Target glucose	Percent time in range between 70 and 180 mg/dL
gluBelow70	Hypoglycemia	Percent time in hypoglycemic range < 70 mg/dL

gluBelow54	Hypoglycemia	Percent time in severe hypoglycemic range < 54 mg/dL
gluAbove180	Hyperglycemia	Percent time in hyperglycemic range > 180 mg/dL
gluAbove250	Hyperglycemia	Percent time in severe hyperglycemic range > 250 mg/dL
gluCV	Glucose variability	Glucose coefficient of variation: (gluSD/gluMean)*100
cgmusestat	Personal CGM	Were participants wearing a personal (non-research) CGM device? [0] no, [1] yes
SevereHypoEvents	Severe hypoglycemic events (lifetime)	Self-reported number of lifetime severe hypoglycemic events. "Severe hypoglycemic event" refers to an episode of low glucose requiring the assistance of another person to treat: [0] 0, [1] 1, [2] 2, [3] 3, [4] 4, [5] 5-10, [6] > 10
SHLast12MonthsB	Severe hypoglycemic events (recent)	Self-reported number of severe hypoglycemic events in the last 12 months. "Severe hypoglycemic event" refers to an episode of low glucose requiring the assistance of another person to treat: [0] 0, [1] 1, [2] > 1
DKANumEverB	Diabetic ketoacidosis	Number of lifetime diabetic ketoacidosis events: [0] 0, [1] 1, [2] 2, [3] 3, [4] 4, [5] 5-10, [6] > 10
DKALast12MonthsB	Diabetic ketoacidosis	Number of diabetic ketoacidosis events in the last 12 months: [0] 0, [1] 1, [2] > 1
microvascular_binary	Microvascular disease	Presence of microvascular disease based on medical record, i.e., retinopathy, nephropathy, neuropathy: [0] no, [1] yes
microvascular_count	Microvascular disease	Number of microvascular diseases based on medical record, i.e., retinopathy, nephropathy, neuropathy: [0-3]
BMI	Body mass index	10,000*(Weight /Height^2), computed based on height and weight measurements from in-person clinic visit
snoring_binary*	Snoring	Snore loudly, measured using STOP-BANG questionnaire for obstructive sleep apnea: [0] no, [1] yes
tired_binary*	Tiredness/fatigue	Tired during the day, measured using STOP-BANG questionnaire for obstructive sleep apnea: [0] no, [1] yes
gasping_binary*	Choking/gasping in sleep	Others have observed gasping during sleep, measured using STOP-BANG questionnaire for obstructive sleep apnea: [0] no, [1] yes
bloodPressure_binary*	Blood pressure	High blood pressure, measured using STOP-BANG questionnaire for obstructive sleep apnea: [0] no, [1] yes
BMI_binary*	Body mass index	BMI > 35 kg/m^2, measured using STOP-BANG questionnaire for obstructive sleep apnea: [0] no, [1] yes
age_binary*	Age	Age > 50 years, measured using STOP-BANG questionnaire for obstructive sleep apnea: [0] no, [1] yes
NeckCir_binary*	Neck circumference	Neck circumference >40 cm, measured using STOP-BANG questionnaire for obstructive sleep apnea: [0] no, [1] yes
gender_male*	Gender	Gender, measured using STOP-BANG questionnaire for obstructive sleep apnea: [0] not male, [1] male
STOP_BANG_sum*	STOP-Bang risk	Continuous risk for obstructive sleep apnea, calculated as the sum of STOP-Bang items (1-point/item x 8-items)
STOP_BANG_risk_num*	STOP-Bang risk	Categorical risk for obstructive sleep apnea (cf. Chung et al. for scoring): [0] low risk, [1] intermediate risk, [2] high risk

* Asterisk identifies items from the STOP-Bang questionnaire for obstructive sleep apnea:

Chung, F., Yegneswaran, B., Liao, P., Chung, S. A., Vairavanathan, S., Islam, S., ... & Shapiro, C. M. (2008). STOP questionnaire: a tool to screen patients for obstructive sleep apnea. *The Journal of the American Society of Anesthesiologists*, 108(5), 812-821.

Supplementary Table 4. Predicting individual differences in average cognition. Six of seven robust predictors of between-person differences in cognitive vulnerability to glucose fluctuations (CV-GF) also explained between-person differences in cognitive performance. Analyses were run using lasso regression (implemented as described in the main text) to predict individual intercepts for DSM RT (u_{0j} in Supplementary Note 1). Below: model performance (mean_RMSE, mean_R2) is shaded gray. Coefficient means and standard deviations (SD) were estimated over *n*=1,000 cross-validation repetitions. N indicates the number of repetitions that retained a given predictor. Bold text identifies robust predictors of between-person differences in cognitive performance (i.e., predictors that were retained in over 50% of repetitions across all EMA cut-offs). Robust predictors of between-person differences in CV-GF (Table 5) are marked by an asterisk (*). Refer to Supplementary Table 3 for codebook. *RMSE = cross-validated root mean squared error; R2 = cross-validated R-squared.*

Variable	50pct: Mean (SD, n)	66pct: Mean (SD, n)	80pct: Mean (SD, n)
mean_RMSE	151.53 (23.38, NA)	155.29 (27.2, NA)	149.9 (28.32, NA)
mean_R2	0.52 (0.01, NA)	0.51 (0.01, NA)	0.5 (0.02, NA)
*** age**	**133.04 (1.2, 1000)**	**136.53 (1.56, 1000)**	**124.47 (3.8, 1000)**
BldPrDia			-6.01 (2.33, 684)
**bloodPressure_binary**	**13.25 (0.12, 1000)**	**12.05 (0.3, 999)**	**17.46 (2.57, 1000)**
DKANumEverB		3.59 (1.24, 986)	3.99 (1.66, 684)
education_num	-6.37 (0.75, 1000)	-5.49 (0.56, 998)	
*** gluBelow70**	**16.41 (0.9, 1000)**	**17.16 (0.99, 999)**	**14.49 (3.85, 999)**
**gluHours**	**-22.02 (1.1, 1000)**	**-19.75 (1.27, 999)**	**-24.5 (3.73, 999)**
gluInRange			-9.01 (4.8, 745)
gluSD			0.62 (0.36, 759)
goToSleepEarliest		2.26 (0.94, 726)	10.87 (5.32, 916)
Height			-8.49 (2.32, 994)
*** microvascular_binary**	**22.56 (0.58, 1000)**	**21.53 (0.9, 999)**	**18.96 (2.85, 999)**
*** NeckCir_binary**	**6.04 (0.25, 1000)**	**7.61 (0.75, 999)**	**11.91 (0.78, 1000)**
NeckCir	1.91 (1.29, 959)		13.8 (6.21, 990)
nReadings			-0.19 (0.03, 999)
**PEHeartRt**	**3.38 (1.31, 998)**	**5.96 (1.24, 996)**	**8.9 (4.83, 844)**
*** SevereHypoEvents**	**18.1 (0.95, 1000)**	**18.62 (0.87, 999)**	**26.43 (2.84, 1000)**
SHLast12MonthsB			2.81 (1.17, 684)
**snoring_binary**	**-14.82 (1.84, 1000)**	**-18.24 (1.94, 999)**	**-18.77 (5.4, 998)**
* tired_binary	0.61 (0.68, 764)		
UnitsInsBasalOrLongAct			-9.86 (4.01, 613)

Supplementary Table 5. Post-hoc tests for Aim 1, H1. Estimates, standard errors (std.error), and 95% credible intervals (conf.low, conf.high) for models examining associations between glucose (centered within-person) and DSM RT in the > 66% completion sample. Cognitive vulnerability to glucose fluctuations was operationalized with respect to quadratic terms for glucose (Glucose^2).

term	estimate	std.error	conf.low	conf.high	group
**66pct_dsm_RT**					
(Intercept)	955.744	17.699	927.802	984.444	NA
Glucose	-472.325	136.098	-695.095	-249.371	NA
Glucose^2	364.977	151.087	120.450	617.038	NA
sd_(Intercept).user_id	225.208	NA	NA	NA	user_id
sd_Glucose. user_id	561.971	NA	NA	NA	user_id
sd_Glucose^2. user_id	338.287	NA	NA	NA	user_id
cor_(Intercept).Glucose.user_id	-0.285	NA	NA	NA	user_id
cor_(Intercept).Glucose^2.user_id	0.320	NA	NA	NA	user_id
cor_Glucose.Glucose^2.user_id	-0.188	NA	NA	NA	user_id
sd_Observation.Residual	124.413	NA	NA	NA	Residual

Supplementary Table 6. Post-hoc tests for Aim 1, H2. Variation in individual estimates of cognitive vulnerability to glucose fluctuations for DSM RT in the >66% completion sample, estimated using post-hoc models that centered (but did not scale) glucose within individuals. Variation was evaluated using the test of practical equivalence. Below: the high-density interval (HDI; [HDI_low, HDI_high]) and region of practical equivalence (ROPE) do not overlap (% inside ROPE = 0.0) with credible interval (CI) = 0.95, indicating statistical significance.

CI	% inside ROPE	HDI_low	HDI_high
0.95	0	7549.699	366184.9

Supplementary Table 7. Post-hoc tests for Aim 2. To evaluate the potential impact of scaling, we ran post-hoc tests for DSM RT in the >66% EMA completion sample. Post-hoc tests centered (but did not scale) glucose within individuals. Model performance (mean_RMSE, mean_R2) is shaded gray. Coefficient means and standard deviations (SD) were estimated over *n*=1,000 cross-validation repetitions. N indicates the number of repetitions that retained a given predictor. Cross-validation sought to minimize mean squared error over lambda values from 4.5 to 7.4 (step size=0.1). This range corresponds to the upper quartile of lambda values that minimized mean squared error in primary analyses, and it retains approximately as many predictors as primary analyses. All seven robust predictors from primary analyses that scaled glucose (marked by *) remained significant in post-hoc analyses that did not scale glucose. Refer to Supplementary Table 3 for codebook. *RMSE = cross-validated root mean squared error; R2 = cross-validated R-squared.*

**Variable**	**Posthoc 66pct: Mean (SD, n)**
mean_RMSE	69.75 (7.2, NA)
mean_R2	0.54 (0, NA)
* age	64.53 (0, 1000)
* microvascular_binary	10.61 (0, 1000)
* gluBelow70	9.77 (0, 1000)
* SevereHypoEvents	8.1 (0, 1000)
* tired_binary	4.56 (0, 1000)
wakeUpTypical	3.55 (0, 1000)
DKANumEverB	3.44 (0, 1000)
NeckCir	3.21 (0, 1000)
bloodPressure_binary	2.84 (0, 1000)
* NeckCir_binary	2.37 (0, 1000)
* gluCV	1.23 (0, 1000)
PEHeartRt	0.8 (0, 1000)
DKALast12MonthsB	0.75 (0, 1000)
SHLast12MonthsB	0.3 (0, 1000)
nReadings	-0.03 (0, 1000)
gluHours	-5.5 (0, 1000)
snoring_binary	-9 (0, 1000)

Supplementary Figure 1. Aim 1, H1: Group associations between glucose and cognitive performance. Clockwise from top left: digit symbol matching (DSM) accuracy, operationalized as number of correct responses; DSM speed, operationalized as median reaction time (RT) in milliseconds (ms) on correct responses; gradual onset continuous performance test (GCPT) speed, operationalized as median RT on correct responses to targets; GCPT accuracy, operationalized as discriminability (d-prime). Point estimates are shaded by EMA completion cut-off (50%, 66%, 80%). Credible intervals (CIs) for linear and quadratic effects are plotted in blue and red, respectively. Thick lines represent 95% CIs, and thin lines represent 90% CIs. Effects were considered significant and marginally significant when 95% and 90% CIs did not overlap zero (dotted black line), respectively. Significant estimates are identified by an asterisk (*), and marginally significant effects are identified by a caret (^).

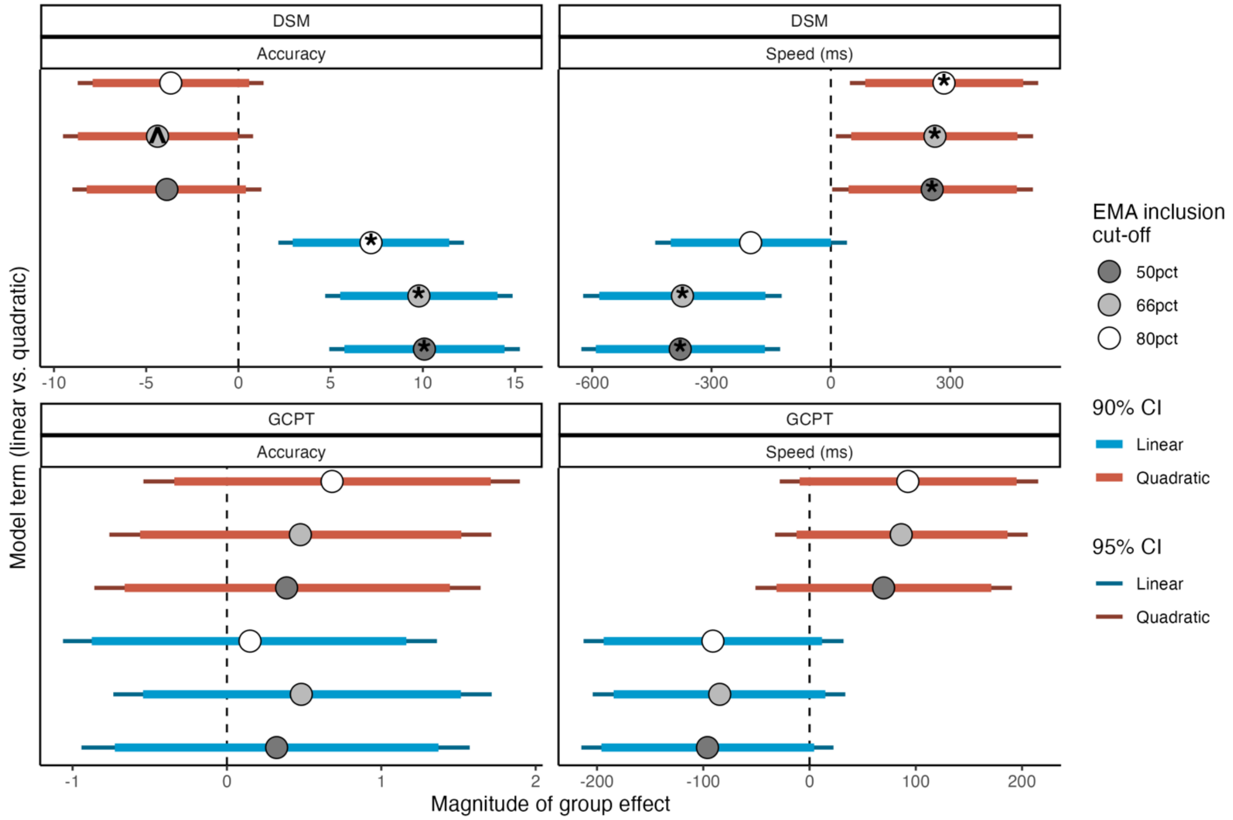

Supplementary Figure 2. Aim 1, H2. Individual estimates of cognitive vulnerability to glucose fluctuations (y-axis) plotted for each participant (x-axis) at different EMA completion cut-offs (panels). Clockwise from top left: gradual onset continuous performance test (GCPT) speed, operationalized as median reaction time (RT) in milliseconds (ms) on correct responses to targets; GCPT accuracy, operationalized as discriminability (d-prime); digit symbol matching (DSM) accuracy, operationalized as number of correct responses; DSM speed, operationalized as median RT on correct responses. Point estimates were computed by summing group and individual coefficients for the quadratic term. Shading identifies 66% (in blue) and 90% (in red) CIs that did not overlap zero.

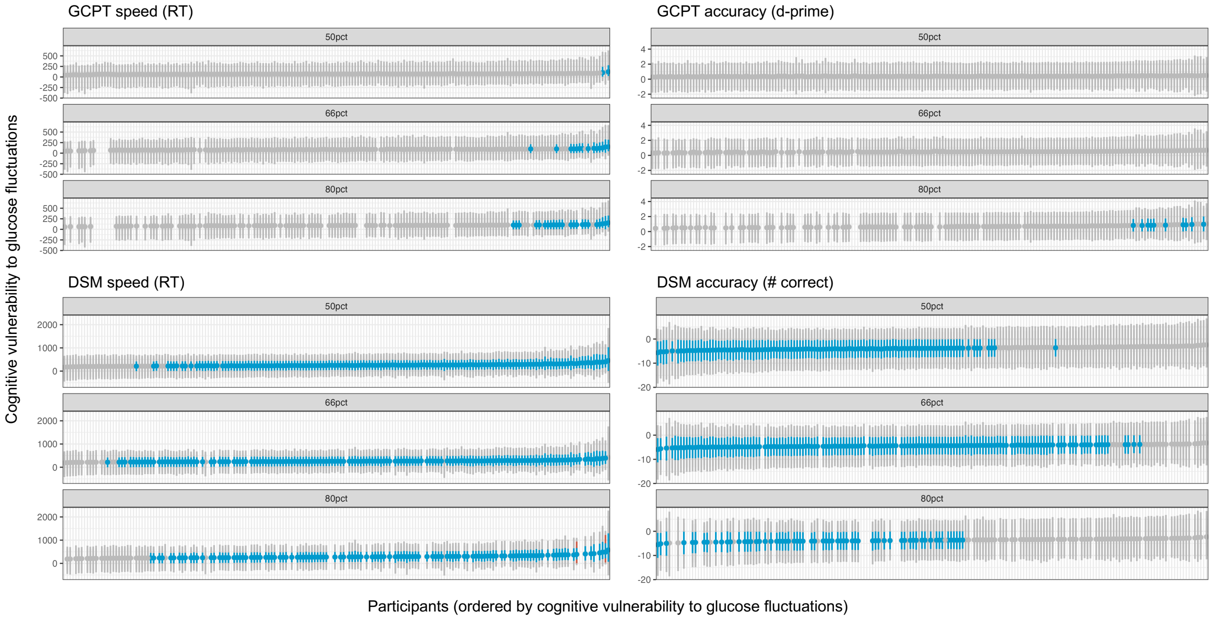

Supplementary Figure 3. Aim 2 correlation matrix. Correlations among independent variables included in data-driven (lasso regression) models.

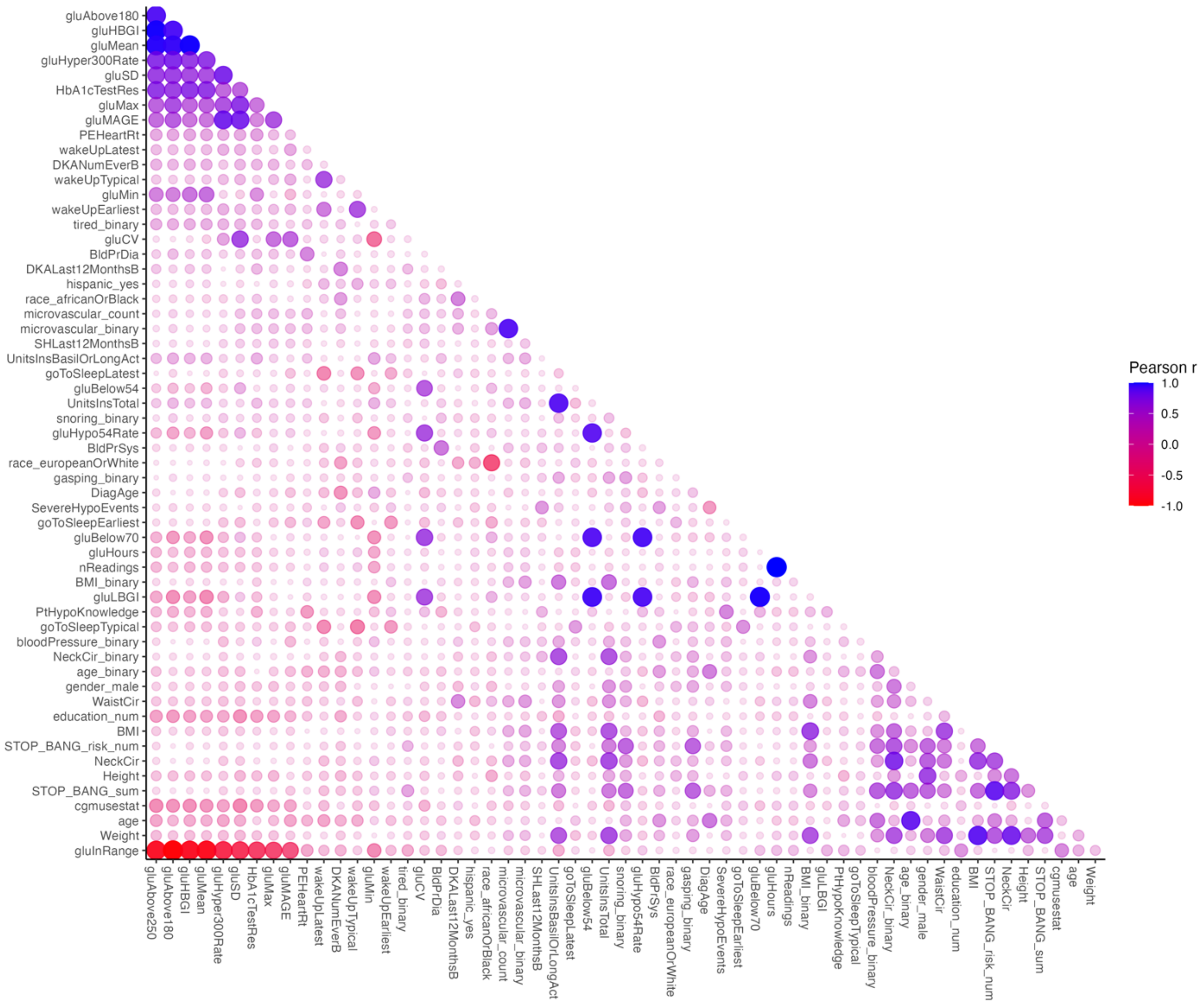

Supplementary Figure 4. Aim 1 variable distributions.

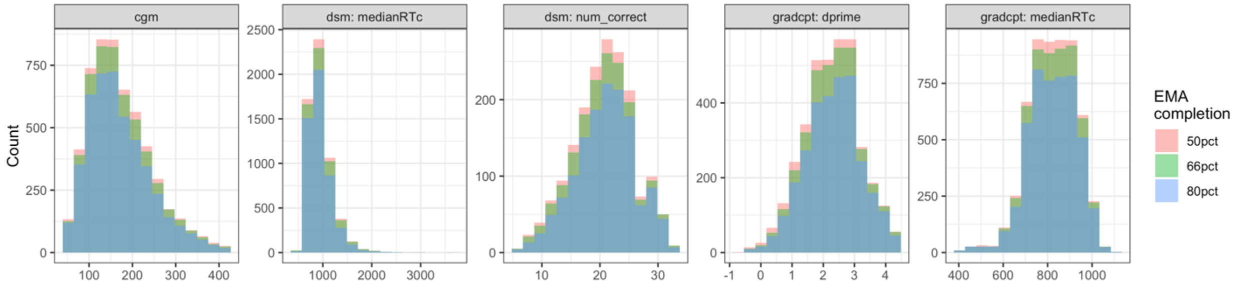

Supplementary Figure 5. Aim 2 variable distributions.

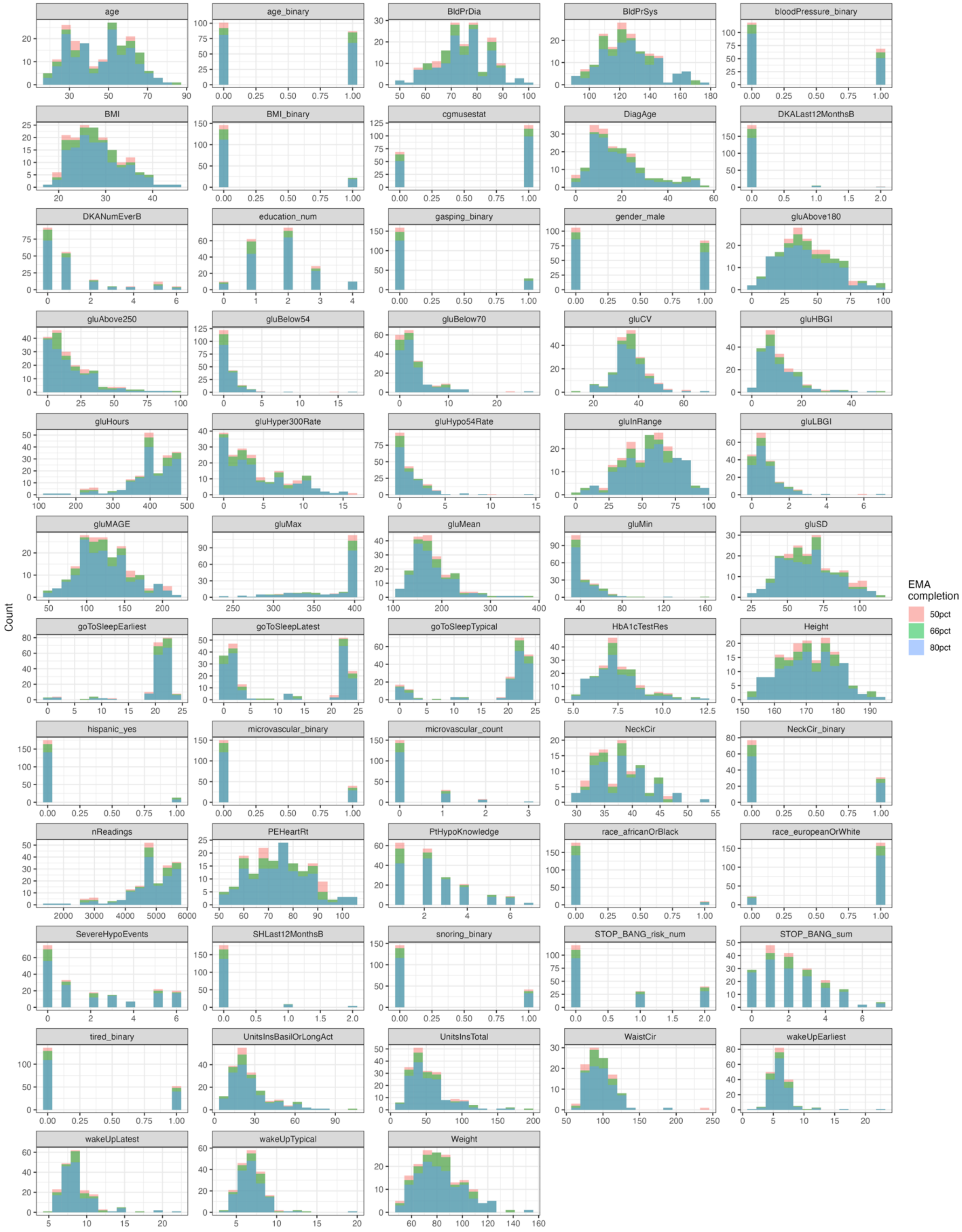

Supplementary Note 1

A. Model form:

Level 1 (observation level *i*):

$$Y_{ij} = \beta_{0j} + \beta_{1j} * glucose_{ij} + \boldsymbol{\beta_{2j}} * glucose_{ij}^2 + \varepsilon_{ij}$$

Level 2 (participant level *j*):

$$\beta_{0j} = \gamma_{00} + u_{0j}$$
$$\beta_{1j} = \gamma_{10} + u_{1j}$$
$$\boldsymbol{\beta_{2j} = \gamma_{20} + u_{2j}}$$

$$Var\begin{bmatrix} u_{0j} \\ u_{1j} \\ \boldsymbol{u_{2j}} \end{bmatrix} = \begin{bmatrix} \tau_{00}^2 & \tau_{01} & \tau_{02} \\ \tau_{10} & \tau_{11}^2 & \tau_{12} \\ \tau_{20} & \tau_{21} & \boldsymbol{\tau_{22}^2} \end{bmatrix}$$

Group (fixed effect) coefficients: γ_{00}, γ_{10}, $\boldsymbol{\gamma_{20}}$
Individual (level 2 random effect) coefficients: u_{0j}, u_{1j}, $\boldsymbol{u_{2j}}$
Standard deviation of individual (level 2 random effect) coefficients: τ_{00}, τ_{11}, $\boldsymbol{\tau_{22}}$
Coefficients relevant to cognitive vulnerability to glucose fluctuations are shaded **gray**

B. Model R syntax:

```
model <-
  stan_lmer(value ~ poly(IV, 2, raw = FALSE) +
              (poly(IV, 2, raw = FALSE) | user_id),
          data = to_model_dsm$data[[1]],
          control=list(adapt_delta=Delta),
          iter = Iterations, chains = 4)
```

C. Model parameters:

EMA cut-off (%)	Outcome	Test	IV	Delta	Iterations
50	DSM	RT	Glu_WP	0.999	15000
50	DSM	Accuracy	Glu_WP	0.999	15000
50	GCPT	RT	Glu_WP	0.999	8000
50	GCPT	Accuracy	Glu_WP	0.999	15000
66	DSM	RT	Glu_WP	0.999	15000
66	DSM	Accuracy	Glu_WP	0.999	20000
66	GCPT	RT	Glu_WP	0.999	8000
66	GCPT	Accuracy	Glu_WP	0.999	15000
80	DSM	RT	Glu_WP	0.999	15000
80	DSM	Accuracy	Glu_WP	0.999	20000

80	GCPT	RT	Glu_WP	0.999	8000
80	GCPT	Accuracy	Glu_WP	0.999	15000
66	DSM	RT	Glu_centered	0.990	20000

EMA = ecological momentary assessment; IV = independent variable; DSM = digit symbol matching; GCPT = gradual onset continuous performance test; RT = reaction time; Glu_WP = glucose, centered and scaled within-person (primary analysis); Glu_centered = glucose, centered within-person (post-hoc analysis)
