# Supplementary material for: Dynamic associations between glucose and ecological momentary cognition in Type 1 Diabetes
Source: NPJ Digit Med. 2024 Mar 18;7:59. doi: 10.1038/s41746-024-01036-5 (PMC10948782; doi:10.1038/s41746-024-01036-5)
Supplement: Supplementary file 2 — Reporting Summary [file 41746_2024_1036_MOESM2_ESM.pdf]

nature portfolio

Corresponding author(s): Zoë Hawks

Last updated by author(s): Jan 24, 2024

Reporting Summary

Nature Portfolio wishes to improve the reproducibility of the work that we publish. This form provides structure for consistency and transparency in reporting. For further information on Nature Portfolio policies, see our Editorial Policies and the Editorial Policy Checklist.

Statistics

For all statistical analyses, confirm that the following items are present in the figure legend, table legend, main text, or Methods section.

n/a	Confirmed	
☐	☒	The exact sample size (*n*) for each experimental group/condition, given as a discrete number and unit of measurement
☐	☒	A statement on whether measurements were taken from distinct samples or whether the same sample was measured repeatedly
☐	☒	The statistical test(s) used AND whether they are one- or two-sided<br>*Only common tests should be described solely by name; describe more complex techniques in the Methods section.*
☐	☒	A description of all covariates tested
☐	☒	A description of any assumptions or corrections, such as tests of normality and adjustment for multiple comparisons
☐	☒	A full description of the statistical parameters including central tendency (e.g. means) or other basic estimates (e.g. regression coefficient) AND variation (e.g. standard deviation) or associated estimates of uncertainty (e.g. confidence intervals)
☐	☒	For null hypothesis testing, the test statistic (e.g. *F*, *t*, *r*) with confidence intervals, effect sizes, degrees of freedom and *P* value noted<br>*Give P values as exact values whenever suitable.*
☐	☒	For Bayesian analysis, information on the choice of priors and Markov chain Monte Carlo settings
☐	☒	For hierarchical and complex designs, identification of the appropriate level for tests and full reporting of outcomes
☐	☒	Estimates of effect sizes (e.g. Cohen's *d*, Pearson's *r*), indicating how they were calculated

Our web collection on statistics for biologists contains articles on many of the points above.

Software and code

Policy information about availability of computer code

Data collection	Links to cognitive EMA tasks are provided in the resource availability statement. Information about the TestMyBrain platform is provided in the manuscript and elaborated in the following publications:<br><br>Treviño, M., Zhu, X., Lu, Y. Y., Scheuer, L. S., Passell, E., Huang, G. C., ... & Horowitz, T. S. (2021). How do we measure attention? Using factor analysis to establish construct validity of neuropsychological tests. Cognitive Research: Principles and Implications, 6(1), 1-26.<br><br>Singh, S., Strong, R. W., Jung, L., Li, F. H., Grinspoon, L., Scheuer, L. S., ... & Germine, L. (2022). The TestMyBrain Digital Neuropsychology Toolkit: Development and Psychometric Characteristics. Journal of clinical and experimental neuropsychology, 1-10.<br><br>Chaytor, N. S., Barbosa-Leiker, C., Germine, L. T., Fonseca, L. M., McPherson, S. M., & Tuttle, K. R. (2021). Construct validity, ecological validity and acceptance of self-administered online neuropsychological assessment in adults. The Clinical Neuropsychologist, 35(1), 148-164.
Data analysis	Analyses were performed in R v4.1.1 using tidyverse for data reduction and visualization, rstanarm and tidybayes for hypothesis-driven (hierarchical Bayesian) modeling, and glmnet for data-driven (lasso) modeling. A link to analysis code is provided in the code availability statement.

For manuscripts utilizing custom algorithms or software that are central to the research but not yet described in published literature, software must be made available to editors and reviewers. We strongly encourage code deposition in a community repository (e.g. GitHub). See the Nature Portfolio guidelines for submitting code & software for further information.

Data

Policy information about availability of data

All manuscripts must include a data availability statement. This statement should provide the following information, where applicable:

- Accession codes, unique identifiers, or web links for publicly available datasets
- A description of any restrictions on data availability
- For clinical datasets or third party data, please ensure that the statement adheres to our policy

Code to analyze and visualize data is available on GitHub (link provided under Code Availability statement in manuscript file). Data are available from the corresponding author on reasonable request (see Data Availability statement in manuscript file).

Research involving human participants, their data, or biological material

Policy information about studies with human participants or human data. See also policy information about sex, gender (identity/presentation), and sexual orientation and race, ethnicity and racism.

Reporting on sex and gender	Gender was self-reported by participants and included among demographic features in data-driven analyses predicting cognitive vulnerability to glucose fluctuations. Descriptive statistics for gender are reported in Table 1 in the manuscript file, and additional information (distributions, codebook) is provided in the supplement. Participant sex was not evaluated.
Reporting on race, ethnicity, or other socially relevant groupings	Education, ethnicity, and race were self-reported by participants and included among demographic features in data-driven analyses predicting cognitive vulnerability to glucose fluctuations. Descriptive statistics are reported in Table 1 in the manuscript file, and additional information (distributions, codebook) is provided in the supplement.
Population characteristics	Participant age, clinical characteristics (hemoglobin A1c, lifetime severe hypoglycemic events), and continuous glucose monitoring descriptive statistics (e.g., mean, standard deviation, coefficient of variation) are provided in Table 1 in the manuscript file. For continuous variables, we report mean, standard deviation, and range. For categorical variables, we report number of observations and percent of observations.
Recruitment	Adults with T1D (N=200, 107 female) were recruited from diabetes and endocrinology centers at Mayo Clinic, State University of New York (SUNY) Upstate Medical University, University of Pennsylvania, and Advent Health. To enroll, participants were required to be over 18 years old, diagnosed with T1D for >1 year, and fluent in English. They were also required to have 24-hour access to a personal smartphone with reliable internet connection, demonstrate understanding of the EMA protocol, and agree to comply with it. Participants were excluded based on the following: inability to complete cognitive assessments owing to significant visual, motor, hearing, or cognitive impairment; any medical or psychiatric condition or treatment that was determined by the principal investigators to interfere with the completion of the study; and inability to complete EMAs (scheduled 9:00 AM-9:00 PM) due to night shift work, planned travel across time zones, and/or other circumstances that would systematically interfere with ability to complete assessments.
Ethics oversight	Written informed consent was obtained prior to enrollment and study procedures were approved by the Jaeb Center for Health Research IRB.

Note that full information on the approval of the study protocol must also be provided in the manuscript.

Field-specific reporting

Please select the one below that is the best fit for your research. If you are not sure, read the appropriate sections before making your selection.

☐ Life sciences ☒ Behavioural & social sciences ☐ Ecological, evolutionary & environmental sciences

For a reference copy of the document with all sections, see nature.com/documents/nr-reporting-summary-flat.pdf

Behavioural & social sciences study design

All studies must disclose on these points even when the disclosure is negative.

Study description	Quantitative, intensive longitudinal, ecological momentary assessment study to characterize dynamic, within-person associations between glucose and cognition in Type 1 Diabetes
Research sample	Adults with Type 1 Diabetes (N=200, 107 female; mean [SD] age = 45.7 [15.6] years) were recruited from diabetes and endocrinology centers at Mayo Clinic, State University of New York (SUNY) Upstate Medical University, University of Pennsylvania, and Advent Health. Exclusion criteria are discussed above (see "Recruitment" subsection), and demographic and clinical characteristics are reported in Table 1. We required participants to have 24-hour access to a smartphone with reliable internet access, which may have introduced sampling bias. Additionally, the sample was predominantly white and non-Hispanic, and future work is needed to confirm generalizability in other samples.
Sampling strategy	Participants were recruited from four adult endocrinology centers with central site coordination by the Jaeb Center for Health

Sampling strategy	Research (JCHR). JCHR has served as a coordinating center for many multi-center clinical trials in Type 1 Diabetes and is recognized internationally as a leader in continuous glucose monitoring research. Sample size (total observations = individuals x observations per individual) was specified in the parent grant award (R01 DK121240). This sample size was selected because it provides good power (80%) for detecting relationships between glycemic variables and cognitive status with R2 as low as 4%. In the present study, we ran analyses across multiple ecological momentary assessment completion cut-offs to ensure robust results.
Data collection	Participants received 45 texts (3 per day x 15 days) prompting them to complete brief (~7-minute) questionnaires and cognitive assessments. Each text corresponded to one ecological momentary assessment (EMA) session, and texts arrived at random times within three 4-hour windows: morning (9:00 AM-12:59 PM), afternoon (1:00-4:59 PM), and evening (5:00-9:00 PM). Time was measured in participants' local time zones. Upon receiving a text message, participants had 30 minutes to start each EMA. If needed, they received a text reminder after 25 minutes had elapsed. Questionnaires were identical within and across EMA sessions (same surveys; surveys presented in the same order). Cognitive assessments were identical within EMA sessions (across participants) but varied across sessions (different test versions; tests presented in different orders). Varying cognitive assessments across sessions discouraged over-reliance on memory, ensuring that tasks remained valid measures of performance within intended cognitive domains. Participants were excluded from data analyses if they completed <50% of EMAs. They received bonus compensation if they completed >80% of EMAs.
Timing	Data were collected between Oct. 2020 and July 2022.
Data exclusions	Continuous glucose monitoring (CGM) data processing: Participants were required to provide >72 hours of raw data from study administered CGM devices. Two participants (1 female) did not provide sufficient CGM data and were excluded from analyses. One participant (female) was excluded due to a protocol deviation (two study-administered CGM devices were worn simultaneously). Within participants, consistent with manufacturer's instructions, we excluded the first 24 hours data from each study-administered device due to reduced accuracy. Exclusions occurred prior to EMA for the first device (inserted during the clinic visit) and around day nine of EMA for the second device (inserted mid-study to replace the first device after 10 days of wear).<br><br>Ecological monetary assessment (EMA) data processing: EMA data were excluded when task performance was comparable to chance or unlikely to occur with adequate and expected effort, based on the following criteria: DSM correct less than 50%, DSM number correct less than six, and GCPT omissions errors greater than 50%. To ensure consistency across participants and sessions, we required responses to be registered using touchscreens, and we required tasks to be marked as complete (e.g., browser did not close prematurely). Participants in the analysis sample provided clean data in at least 50% of possible EMA sessions. Five participants (all male) were excluded because they did not meet these quality control criteria in 50% or more of possible EMA sessions. Two participants (1 female) were excluded due to a technical anomaly that allowed them to complete more than 45 EMA sessions.<br><br>After CGM + EMA processing: the analysis sample (n=190) did not differ from the full sample (N=200) with respect to any of the following: age, gender, race, ethnicity, educational attainment, hemoglobin A1c, CGM summary statistics (glucose mean, standard deviation, coefficient of variation), CGM percent time in range (70-180 mg/dL, below 70 mg/dL, below 54 mg/dL, above 180 mg/dL, above 250 mg/dL), or number of lifetime severe hypoglycemic events (ps > .05).
Non-participation	N = 14 participants dropped out or declined participation for the following reasons: never started at clinic or reassigned user id number (3), technical problems before reaching 50% EMA completion (1), elected to discontinue before reaching 50% EMA completion (2), protocol non-compliance, e.g., consistent failure to complete EMAs (8)
Randomization	Participants were not allocated into experimental groups.

Reporting for specific materials, systems and methods

We require information from authors about some types of materials, experimental systems and methods used in many studies. Here, indicate whether each material, system or method listed is relevant to your study. If you are not sure if a list item applies to your research, read the appropriate section before selecting a response.

Materials & experimental systems

n/a	Involved in the study
☒	☐ Antibodies
☒	☐ Eukaryotic cell lines
☒	☐ Palaeontology and archaeology
☒	☐ Animals and other organisms
☒	☐ Clinical data
☒	☐ Dual use research of concern
☒	☐ Plants

Methods

n/a	Involved in the study
☒	☐ ChIP-seq
☒	☐ Flow cytometry
☒	☐ MRI-based neuroimaging
